# Self-management support interventions to reduce health care utilisation without compromising outcomes: a systematic review and meta-analysis

**DOI:** 10.1186/1472-6963-14-356

**Published:** 2014-08-27

**Authors:** Maria Panagioti, Gerry Richardson, Nicola Small, Elizabeth Murray, Anne Rogers, Anne Kennedy, Stanton Newman, Peter Bower

**Affiliations:** School for Primary Care Research, Centre for Primary Care, Institute of Population Health, University of Manchester, Williamson Building, Oxford Road, Manchester, M13 9PL UK; Centre for Health Economics, University of York, Heslington, York, YO10 5DD UK; Research Department of Primary Care and Population Health, University College London, Rowland Hill Street, London, NW3 2PF UK; Faculty of Health Sciences, University of Southampton, Highfield, Southampton, SO17 1BJ UK; School of Health Sciences, City University London, 1 Myddleton Street, London, EC1V 0HB UK

**Keywords:** Self-management support interventions, Long-term conditions, Health outcomes, Quality of life, Health care utilization, Hospitalizations, Costs, Cost-effectiveness, Systematic review, Meta-analysis

## Abstract

**Background:**

There is increasing interest in the role of ‘self-management’ interventions to support the management of long-term conditions in health service settings. Self-management may include patient education, support for decision-making, self-monitoring and psychological and social support. Self-management support has potential to improve the efficiency of health services by reducing other forms of utilisation (such as primary care or hospital use), but a shift to self-management may lead to negative outcomes, such as patients who feel more anxious about their health, are less able to cope, or who receive worse quality of care, all of which may impact on their health and quality of life. We sought to determine which models of self-management support are associated with significant reductions in health services utilisation without compromising outcomes among patients with long-term conditions.

**Methods:**

We used systematic review with meta-analysis. We included randomised controlled trials in patients with long-term conditions which included self-management support interventions and reported measures of service utilisation or costs, as well as measures of health outcomes (standardized disease specific quality of life, generic quality of life, or depression/anxiety).We searched multiple databases (CENTRAL, CINAHL, Econlit, EMBASE, HEED, MEDLINE, NHS EED and PsycINFO) and the reference lists of published reviews. We calculated effects sizes for both outcomes and costs, and presented the results in permutation plots, as well as conventional meta-analyses.

**Results:**

We included 184 studies. Self-management support was associated with small but significant improvements in health outcomes, with the best evidence of effectiveness in patients with diabetic, respiratory, cardiovascular and mental health conditions. Only a minority of self-management support interventions reported reductions in health care utilisation in association with decrements in health. Evidence for reductions in utilisation associated with self-management support was strongest in respiratory and cardiovascular problems. Studies at higher risk of bias were more likely to report benefits.

**Conclusions:**

Self-management support interventions can reduce health service utilization without compromising patient health outcomes, although effects were generally small, and the evidence was strongest in respiratory and cardiovascular disorders. Further work is needed to determine which components of self-management support are most effective.

**Electronic supplementary material:**

The online version of this article (doi:10.1186/1472-6963-14-356) contains supplementary material, which is available to authorized users.

## Background

With the increasing prevalence of long-term conditions [1], and with many patients reporting multimorbidity [2], there is worldwide interest in innovations in service delivery that can better manage these patients [3]. The global financial crisis and subsequent constraint on spending has led to a shift in focus to more efficient models of care which can reduce high cost service use such as emergency hospital admissions. This has in turn led to a focus on patients at high risk of high utilisation, with the introduction of algorithms to identify those patients and complex case management interventions to manage them. However, evidence of the effectiveness of this approach is limited [4], and recent commentators have highlighted that a focus on very high risk cases limits impact, because they account for only a small proportion of overall health care use [5].

This has increased interest in the wider group of patients with long-term conditions. It has been suggested that many patients with long-term conditions can be managed effectively by effective support for ‘self-management’. Self-management has been defined as ‘the care taken by individuals towards their own health and well being: it comprises the actions they take to lead a healthy lifestyle; to meet their social, emotional and psychological needs; to care for their long-term condition; and to prevent further illness or accidents’, and can include responding to symptoms, managing acute episodes, relaxation, exercise and smoking cessation, managing the emotional impact of conditions, and working effectively with health professionals and other community resources [6]. Self-management support in the United Kingdom National Health Service is provided through various platforms, including increasing access to health information [7], deployment of assistive technologies such as telehealth and telecare [8, 9]; community based skills-training and support networks [10–12], and interventions led by health professionals [13].

### Self-management and demand management

A key driver of the interest in self-management is the potential to make a significant contribution to *efficient* health care delivery [14], by increasing patient engagement in care, improving uptake of preventive activities, and reducing reliance on formal health care services by better management of existing conditions. However, the scale of the contribution of self-management to the management of demand is unclear. Positive reports of impacts of self-management support on health care utilisation [15] have not always been replicated [16], and some self-management interventions may increase demand [17].

**Figure 1:**
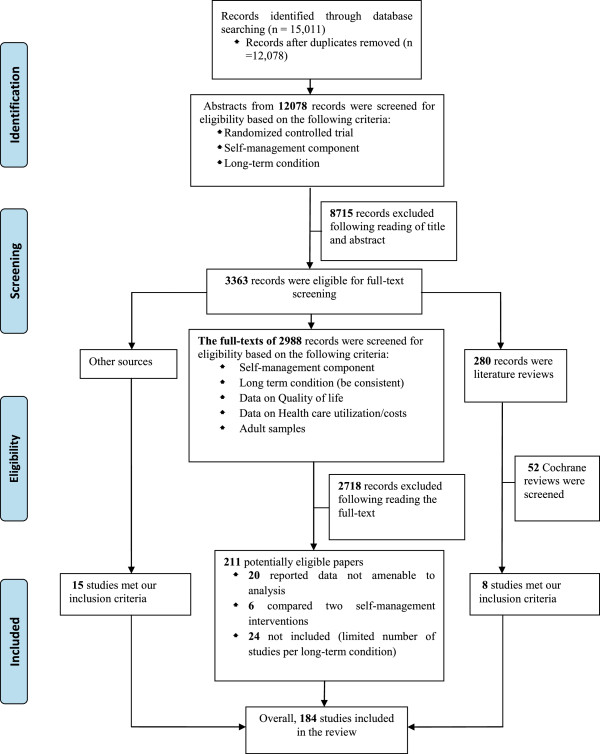
**PRISMA flow diagram.**

In economic terms, efficiency involves maximising outcomes for a given cost or minimising costs for a given level of outcome. When interventions improve outcomes and increase costs (see Figure 1), decision-makers are faced with decisions about ‘allocative efficiency’, where additional resources are needed for a new service, which may incur opportunity costs for other groups of patients [18]. However, in the context of financial pressures, there may be equal interest in the interventions which are less costly and at least as effective as current treatments (known as ‘technically efficient’ interventions) [18]. There is an implicit assumption that self-management support has the potential to be technically efficient. This may occur by patients undertaking care traditionally done by health professionals (for example, monitoring of blood pressure), or by better management of long-term conditions, which enables complications and crises (and subsequent hospital admission) to be avoided.

However, increasing self-management is not uncomplicated. If patients are poorly prepared for new roles, they may suffer negative outcomes. Increasing the role of patients in the management of long-term conditions, while reducing access to formal services, may result in anxiety, coping difficulties, and reduced quality of care. Achieving cost savings while significantly reducing patient quality of life would be a poor outcome for health services and patients.

The research question underlying this review was: What models of self-management support are associated with significant reductions in health services utilisation without compromising outcomes, among patients with long-term conditions?

## Methods

The review protocol is available as part of the PROSPERO database (registration number: CRD42012002694). Minor deviations from the published protocol were made in data extraction procedures because of the large number of studies identified by the review.

### Population

We included studies of patients with a long-term condition, defined generically as ‘one that cannot be cured but can be managed through medication and/or therapy.’ This included common physical conditions such as diabetes, asthma, coronary heart disease, and mental health conditions such as depression. We also included studies recruiting patients with a mix of long-term conditions, and those recruiting on the basis of multimorbidity. We excluded subjects under 18 years of age, and studies conducted in the developing world.

### Intervention

For the purposes of the review, we defined a self-management support intervention as:

‘An intervention primarily designed to develop the abilities of patients to undertake management of health conditions through education, training and support to develop patient knowledge, skills or psychological and social resources’.

We included all formats and delivery methods (group or individual, face to face or remote, professional or peer led). We included all studies which included a significant component of self-management support. After initial screening of a proportion of the studies, we distinguished 2 categories:

- ‘Self-management’ including provision of materials and support from a health professional or trained peer. We coded the amount of support as in three categories for descriptive purposes: ‘pure’ self-management (no support), ‘supported self-management’ (up to 2 hours of additional support for the total durationof the study) and ‘intensively supported self-management’ (more than 2 hours of additional support).
- ‘Case management’ (with more than 2 hours of additional support, and including input from a multidisciplinary team).

Two authors independently assessed the type of intervention and disagreements were resolved via discussion.

### Comparisons

We included studies where the self-management support intervention was compared against usual care alone, or where the self-management support intervention was compared against a more intensive ‘usual care’ intervention (e.g. self-management versus conventional hospital use). We excluded studies where two versions of self-management support interventions were compared as such comparisons did not allow assessment of the impact of the self-management support per se.

### Outcomes

We extracted data on core types of health care utilisation, with a focus on comprehensive measures (i.e. cost summaries including multiple sources of utilisation) or major cost drivers (i.e. hospital use). Other, more minor costs (such as medication and primary care visits) were identified but not analysed. We also separately extracted data on outcomes relating to patient quality of life and health outcomes, including standardised measures of disease specific quality of life outcomes, generic quality of life, and depression/anxiety. We excluded measures of psychological or clinical variables which did not provide a direct assessment of health or quality of life, such as self-management behaviour, self-efficacy, HbA1C or forced expiratory volume (FEV), as these are likely to be unreliable indicators of health related quality of life [19].

### Study design

We restricted the review to randomised controlled trials.

### Identification of studies

We conducted a primary search of multiple databases in June 2012. Databases included the CENTRAL register of controlled trials, CINAHL, Econlit, EMBASE, HEED, MEDLINE, MEDLINE in process, NHS EED and PsycINFO. An example search is listed in Additional file 1. We also identified eligible studies by checking published reviews (listed in Additional file 1).

Titles and abstracts were screened for eligibility. More than 40% of titles and abstracts (n = 5,000) were screened by 2 researchers independently (kappa = 0.87). Screening of the remaining titles and abstracts was completed by one reviewer. Approximately one third of the full texts were screened by 2 reviewers independently (kappa = 0.85), with the remaining full-texts screened by one reviewer. The studies had to fulfil five inclusion criteria to be eligible for inclusion in the review:

- Randomised controlled trial
- Diagnosis of a long-term condition
- Self-management or case management intervention
- Adults (aged ≥ 18 years)
- Report quantitative data on health care utilization (hospitalization rates/costs and total costs) and health outcomes (quality of life, depression and anxiety). Studies reporting non-amenable data for meta-analysis on both, health outcomes and health care utilization, were excluded from the review.

### Data extraction

Descriptive data on studies, populations and interventions were extracted by 2 researchers working independently. A subset of data on quantitative outcomes (n = 50) were extracted by 2 members of the research team working independently, with the rest of the data extracted by one member and checked by a second.

We extracted data on the effect of self-management interventions on health care utilisation and total costs. To assess study quality, we chose a dichotomous measure based on allocation concealment, as this is consistently associated with treatment effect [20, 21]. Allocation concealment was judged as adequate or inadequate according to the Cochrane risk of bias tool. We analysed outcomes, grouping by risk of bias to assess whether results varied by study quality.

### Analyses

We calculated standardised mean differences for health outcomes and costs using reported data or appropriate transformation and imputation [22]. Some measures of utilisation (e.g. hospital length of stay) and costs demonstrate significant skew. In line with published reviews, we identified those outcomes where the standard deviation multiplied by two was greater than the mean, as this indicates that the mean is not a good indicator of the centre of the distribution [23, 24], although skewed data are less problematic if the sample size is large. Cluster trials were identified and the precision of analyses adjusted using a sample size/variation inflation method [25], assuming an intra-class correlation of 0.02. Studies reporting multiple self-management interventions were treated as separate comparisons, with appropriate adjustment of sample sizes to avoid double counting. We explored statistical heterogeneity through the I^2^ statistic [26], labelling levels of heterogeneity as ‘low’ (0%-25%), ‘moderate’ (26-74%) and ‘high’ (75%+).

We present the results of the included studies according to a permutation plot [27]. This involves plotting the effect of interventions on utilisation and outcomes simultaneously and placing them in quadrants of the cost effectiveness plane depending on the pattern of outcomes. Such plots identify studies in the appropriate quadrant (i.e. those that reduce costs without compromising outcomes) and those in problematic quadrants (i.e. those that reduce costs but also compromise outcomes, or those that compromise both outcomes and costs).

Hospital use generally represents a significant driver of total costs, but limiting analysis to a single cost source leaves the analysis vulnerable to cost shifting, where benefits found in terms of reductions in hospital use mask increases in costs elsewhere (e.g. to primary care, or patient out of pocket costs). We presented two permutation plots, one based on studies reporting a measure related to hospital use, and one based on total costs.

For each condition, we conducted separate meta-analyses of the effects of self-management interventions in trials reporting utilisation outcomes (separately for total costs and hospital use outcomes) and in trials reporting health outcomes. We conducted secondary analyses, exploring differences by study quality (high and low risk of bias) and country of origin (UK versus non-UK).

As a secondary analysis, we then identified the subset of trials of self-management interventions reporting *both* utilisation and health outcomes, and conducted a meta-analysis of the effects of self-management interventions on utilisation and health outcomes, in this subset of trials. We conducted these sensitivity analyses in those long-term conditions where there were at least 10 studies with both outcomes. We repeated each of these analyses, distinguishing self-management and case management, as defined previously.

We created funnel plots [28] using standard errors [29] (with associated regression tests) to assess the potential for small sample bias for each outcome.

## Results

### Study characteristics

The PRISMA diagram is shown in Figure 1, with study references listed in Additional file 1. A completed PRISMA checklist is listed in Additional file 2. Descriptive characteristics of the studies are provided in Table 1.

**Table 1:** **Basic descriptive data on the studies**

Category	Characteristics	N (n = 184)
Context	Country	
	UK	43 (23%)
	US	65 (35%)
	European	44 (24%)
	Other	32 (17%)
Patients	Condition	
	Arthritis	14 (8%)
	Cardiovascular	53 (29%)
	Diabetes	11 (6%)
	Mental health	29 (16%)
	Mixed disease	13 (7%)
	Respiratory	44 (24%)
	Pain	20 (11%)
	Mean Age (SD)	58 (13)
	% Male	49%
Intervention	Content	
	Pure SM	9 (5%)
	Supported SM	36 (20%)
	Intensive SM	87 (47%)
	Case management	52 (28%)
	Sample size (SD)	275 (272)
	Range	23-1801
External validity	Excluded patients with other long-term conditions	65 (35%)
	Proportion of eligible patients who did not take part in the study	
	Not clear	48 (26%)
	<20%	40 (22%)
	21-40%	55 (30%)
	41-60%	25 (14%)
	61-80%	14 (8%)
	81-100%	2 (1%)

### Relationships between cost and health outcomes

**Figure 2:**
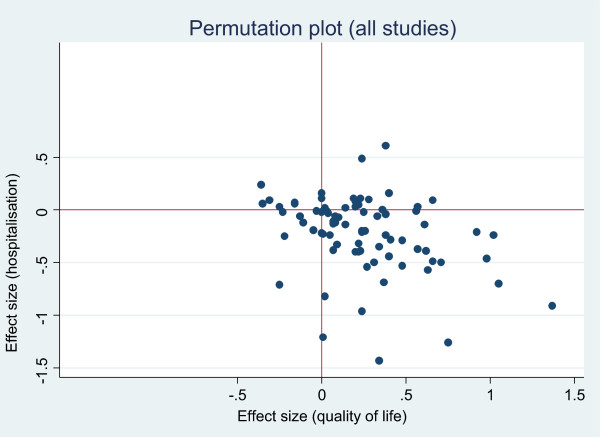
**Permutation plot – health outcomes and hospital use outcomes.**

**Figure 3:**
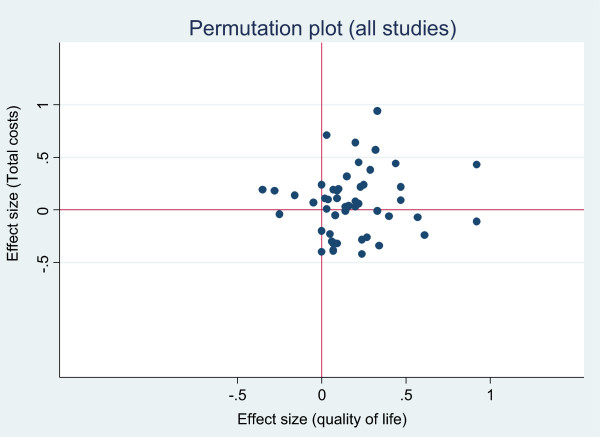
**Permutation plot – health outcomes and total costs.**

Figures 2 and 3 show the overall permutation plots, plotting health outcomes and hospital use outcomes (Figure 2) and health outcomes and costs (Figure 3). In terms of hospital use, the bulk of studies are in the lower right quadrant (i.e. they are associated with improvements in health outcomes and reductions in utilisation). Only a minority of studies report decrements in health outcomes, and a smaller proportion of studies report improved outcomes with increases in utilisation. In terms of costs, the picture is more mixed, with more studies in the top right quadrant, reporting improved outcomes with increases in costs. Note that the plots do not represent the uncertainty around point estimates, which in many studies would be considerable. Of the 71 studies reporting costs, almost all demonstrated significant skew (i.e. the standard deviation multiplied by 2 was more than twice the mean).

### Effects of self-management support on health outcomes and utilisation

Table 2 shows the impact of self-management support on hospital use and health outcomes, structured by type of long-term condition and type of self-management support. Results are highlighted in the table that show an effect size of 0.2 (at least a ‘small’ effect by current convention), where the effect is also statistically significant. As can be seen from Table 2, such impacts are found in a number of cells in relation to health outcomes, but are restricted to interventions in respiratory and cardiovascular populations in relation to hospital use.

**Table 2:** **Summary – hospital use (overall ES, 95% CI, N, I** ^**2**^ **)**

	Combined QoL	Self-management QoL	Case management QoL	Combined hospital use	Self-management hospital use	Case management hospital use
**Respiratory**	**0.27 (0.16 to 0.37, n = 34, moderate)**	**0.28 (0.16 to 0.41, n = 27, moderate)**	0.19 (0.02 to 0.36, n = 7, low)	**−0.21 (−0.32 to −0.09, n = 31, moderate)**	−0.19 (−0.33 to −0.05, n = 25, moderate)	**−0.26 (−0.42 to −0.10, n = 6, zero)**
**Cardiac**	**0.21 (0.14 to 0.28, n = 40, moderate)**	0.19 (0.10 to 0.27, n = 27, moderate)	**0.26 (0.12 to 0.39, n = 13, moderate)**	**−0.23 (−0.34 to −0.13, n = 38, high)**	**−0.20 (−0.33 to −0.07, n = 25, high)**	**−0.29 (−0.47 to −0.11, n = 13, high)**
**Arthritis**	0.16 (0.07 to 0.26, n = 11, zero)	0.17 (0.07 to 0.27, n = 7, zero)	0.13 (−0.13 to 0.39, n = 4, zero)	−0.06 (−0.22 to 0.10, n = 6, moderate)	−0.02 (−0.19 to 0.16, n = 5, moderate)	**−0.24 (−0.48 to 0.00, n = 1, NA)**
**Pain**	0.13 (0.04 to 0.21, n = 19, low)	0.12 (0.02 to 0.22, n = 15, low)	0.20 (−0.10 to 0.50, n = 4, zero)	−0.03 (−0.34 to 0.28, n = 3, low)		−0.03 (−0.34 to 0.28, n = 3, low)
**Diabetes**	**0.44 (0.14 to 0.75, n = 10, high)**	**0.44 (0.14 to 0.75, n = 10, high)**		−0.12 −0.29 to 0.05, n = 5, moderate)	−0.12 −0.29 to 0.05 n = 5, moderate)	
**Mental health**	**0.22 (0.11 to 0.33, n = 26, high)**	0.05 (−0.07 to 0.17, n = 15, moderate)	**0.38 (0.24 to 0.51, n = 11, high)**	−0.03 (−0.10 to 0.04, n = 21, low)	−0.03 (−0.16 to 0.10, n = 13, moderate)	−0.04 (−0.13 to 0.05, n = 8, zero)
**Mixed**	0.13 (0.02 to 0.24, n = 10, moderate)	0.11 (−0.03 to 0.24, n = 7, moderate)	0.22 (−0.03 to 0.48, n = 3, moderate)	−0.12 (−0.20 to −0.03, n = 11, moderate)	−0.09 (−0.17 to −0.02, n = 8, zero)	−0.13 (−0.40 to 0.14, n = 3, moderate)

Bold letters: > =0.2 and statistically significant effects.

Table 3 is structured in the same way, but details the impact of self-management support on costs and health outcomes. The patterns are broadly similar, although effects are now reported for arthritis and pain, but restricted to case management interventions. It should be noted that some of the differences between Tables 2 and 3 reflect changes in the number of studies included in the analysis and associated precision of the estimates.

**Table 3:** **Summary – costs (overall ES, 95% CI, N, I** ^**2**^ **)**

	Combined QoL	Self-management QoL	Case management QoL	Combined costs	Self-management costs	Case management costs
**Respiratory**	**0.27 (0.16 to 0.37, n = 34, moderate)**	**0.28 (0.16 to 0.41, n = 27, moderate)**	0.19 (0.02 to 0.36, n = 7, low)	0.09 (−0.14 to 0.33, n = 9, high)	0.09 (−0.19, 0.37) N = 6, high)	0.09 (−0.46 to 0.64, n = 3, high)
**Cardiac**	**0.21 (0.14 to 0.28, n = 40, moderate)**	0.19 (0.11 to 0.27, n = 27, moderate)	**0.26 (0.12 to 0.39, n = 13, moderate)**	**−0.25 (−0.47 to −0.04, n = 9, moderate)**	−0.25 (−0.82, 0.32, n = 4, high)	**−0.27 (−0.44, −0.10, n = 5, moderate)**
**Arthritis**	0.16 (0.07 to 0.26, n = 11, zero)	0.17 (0.07 to 0.27, n = 7, zero)	0.13 (−0.13 to 0.39, n = 4, zero)	0.07 (−0.07 to 0.20, n = 11, moderate)	0.14 (0.01 to 0.27, n = 8, moderate)	**−0.28 (−0.53 to −0.03, n = 3, zero)**
**Pain**	0.13 (0.04 to 0.21, n = 19, zero)	0.12 (0.02 to 0.22, n = 15, low)	0.20 (−0.11 to 0.51, n = 4, zero)	0.07 (−0.13 to 0.28, n = 13, high)	0.15 (−0.06 to 0.36, n = 11, high)	**−0.41 (−0.74 to −0.08), n = 2, zero)**
**Diabetes**	**0.44 (0.14 to 0.75, n = 10, high)**	**0.44 (0.14 to 0.75, n = 10, high)**		0.19 (−0.18, 0.55, n = 4, moderate)	0.19 (−0.18, 0.55, n = 4, moderate)	
**Mental health**	**0.22 (0.11 to 0.33, n = 26, high)**	0.05 (−0.07 to 0.17, n = 15, moderate)	**0.38 (0.24 to 0.51, n = 11, high)**	0.03 (−0.05 to 0.11, n = 14, low)	−0.04 (−0.23 to 0.15, n = 4, moderate)	0.05 (−0.04 to 0.13, n = 10, low)
**Mixed**	0.13 (0.02 to 0.24, n = 10, moderate)	0.11 (−0.03 to 0.24, n = 7, moderate)	0.22 (−0.03 to 0.48, n = 3, low)	0.06 (−0.02 to 0.13, n = 7, zero)	0.05 (−0.04 to 0.13, n = 6, zero)	0.11 (−0.09 to 0.31, n = 1, N/A)

Bold letters: > =0.2 and statistically significant effects.

The sensitivity analyses (not shown) showed similar patterns of results when analyses are restricted to the subset of studies which report both health outcomes and utilisation/cost data.

### Study quality and small study bias

Table 4 shows the effects of self-management support on the three core outcomes, grouped according to risk of bias. Studies judged at high risk of bias reported greater benefits in health outcomes and greater reductions in hospitalization than those judged at low risk of bias, although they were also associated with increases in total costs. Table 5 shows the effects of self-management support on the three core outcomes, grouped according to country of origin (UK or other). The results suggest that studies in the UK demonstrated smaller effects on health outcomes. Conversely, studies in the UK demonstrated larger reductions in hospitalisation, but those were not matched by cost data, where UK studies showed a moderate increase in overall costs.

**Figure 4:**
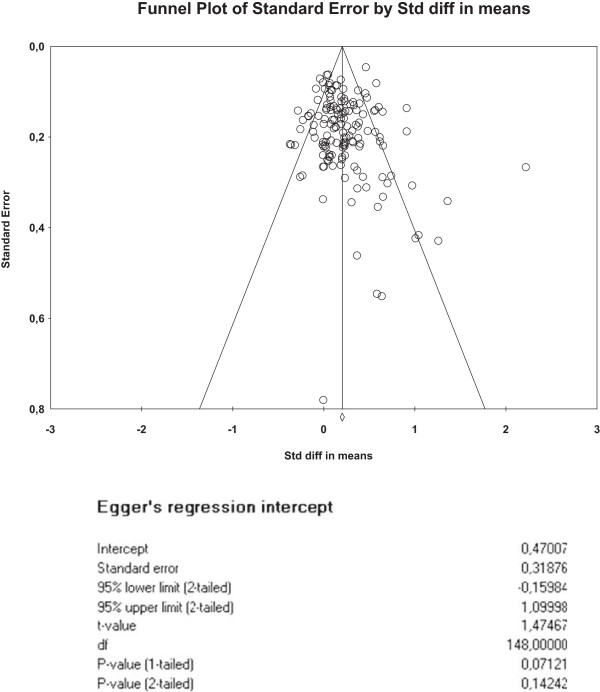
**Funnel plot – health outcomes.**

**Figure 5:**
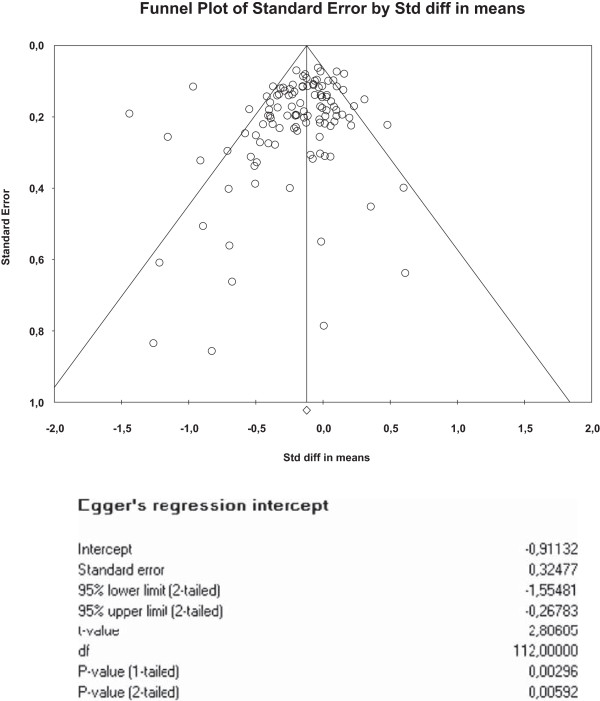
**Funnel plot – hospital use.**

**Figure 6:**
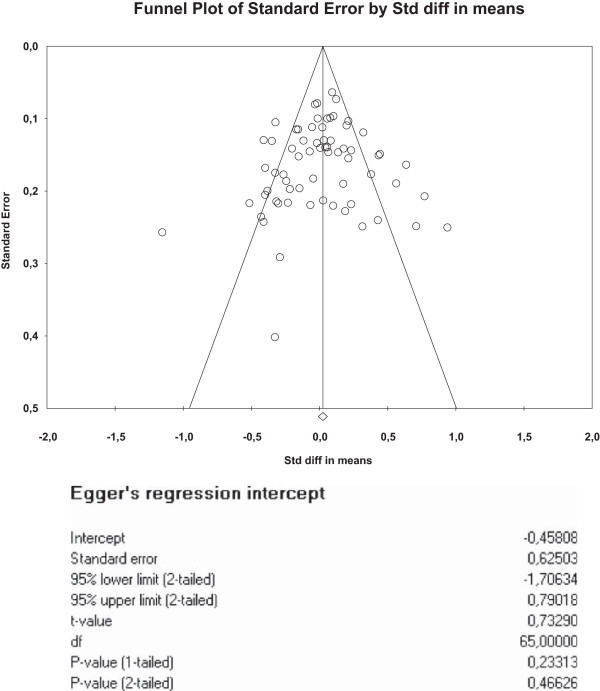
**Funnel plot – total costs.**

The funnel plots for health outcomes (intercept 0.47, 95% CI −0.16 to 1.10, p = 0.14) (Figure 4) and costs (intercept −0.46, 95% CI −1.71 to 0.79, p = 0.47) (Figure 5) did not show evidence of small study bias. The plot for hospital use (intercept −0.91, 95% CI −1.55 to −0.27, p = 0.01) did show evidence of small study bias (Figure 6).

**Table 4:** **Overall effects by risk of bias**

	Overall effect size	Effect size (high risk of bias)	Effect size (low risk of bias)
	95% CI	95% CI	95% CI
	I ^2^	I ^2^	I ^2^
**Health outcomes**	0.22 (0.17 to 0.26)	0.23 (0.18 to 0.29)	0.18 (0.12 to 0.25)
**Hospital use**	−0.16 (−0.20 to −0.11)	−0.18 (−0.24 to −0.11)	−0.10 (−0.16 to −0.04)
**Costs**	0.02 (−0.05 to 0.08)	0.07 (−0.05 to 0.18)	−0.01 (−0.09 to -0.07)

**Table 5:** **Overall effects by country**

	Overall effect size	Effect size (UK studies)	Effect size (Non UK studies)
**Health outcomes**	0.22 (0.17 to 0.26)	0.10 (0.05 to 0.14)	0.25 (0.19 to 0.30)
**Hospital use**	−0.16 (−0.20 to −0.11)	−0.23 (−0.35 to −0.11)	−0.14 (−0.19 to −0.09)
**Costs**	0.02 (−0.05 to 0.08)	0.13 (0.02 to 0.24)	−0.04 (−0.12 to 0.04)

## Discussion

In summary, self-management support interventions generally had a small but positive impact on health outcomes, with only a small minority of studies included in the review reporting decrements in outcomes in the permutation plots. In terms of the primary utilisation outcome of hospital use, the evidence was most robust in both scope and effect in relation to interventions in respiratory and cardiovascular problems. The magnitude of those effects was similar in cost outcomes.

### Strengths and limitations

The study was conducted and reported in line with current guidance. The high number of included studies and the rapid timeframe of the review meant that we could not use 2 independent researchers for all assessments and extraction, but we tested the reliability and found high levels of agreement.

Designing searches and inclusion criteria for self-management is complex, because of the lack of consensus on the scope of the term. Our search was broad, but was dependent on the existence of key terms in the titles and abstracts. Additionally, it is not clear how the search terms for utilisation or other economic outcomes perform in terms of sensitivity or specificity, although they were tested against a known set of studies. We did not find evidence of publication bias in relation to health outcomes or costs, but there was evidence in terms of hospital use data.

The broad scope of the review combined with the large number of studies meant that a less comprehensive quality assessment was used. This does mean that quality assessment was very dependent on the exact descriptions of concealment provided in the papers, and the more limited quality assessment may not be as reliable as a full risk of bias assessment.

We required that data were reported in a way that was amenable to meta-analysis for cost and health outcomes. Such selection would potentially cause selection effects in the included studies. We were unable to formally test differences between eligible studies reporting or not reporting appropriate data, as data on the latter were not extracted because of resource limitations, and would by definition not have allowed assessment of variation in *outcomes*.

Our aim (to identify studies that reduce costs while not compromising outcomes) reflects the current economic context faced by many commissioners of health care services, but does not map neatly onto current economic analyses, which focus on the incremental cost effectiveness ratio and net mean benefit. Self-management interventions which reduce costs without compromising outcomes might be attractive to commissioners, but so might interventions which increase costs, while providing significant additional health benefits.

The most comprehensive assessment of costs would include all those related to the intervention (NHS services, social care and other costs, patient direct costs and costs of lost productivity). However, such comprehensive costing is rare, and more limited assessments of utilisation still have utility, as some forms of utilisation (such as hospital costs) are generally a major driver of total costs. However, caution must be exercised in interpretation of studies reporting partial cost data, as there is always the danger of cost shifting rather than genuine reduction (as evidenced in the comparison of Figures 2 and 3). Comprehensive costing will include the costs of the self-management intervention itself which is required to generate reductions in hospital use.

Multimorbidity is prevalent among patients with long-term conditions, but a recent review of interventions found few studies [30], and our main analysis has been in terms of disease categories. It is possible that the results reported here could be significantly moderated by multimorbidity [31]. Our analysis assumed that self-management support is a ‘health technology’ which is potentially discrete, defined, and capable of being delivered in a standardised form. Clearly, many aspects of self-management are not of this type (such as that provided within social networks and informal care) [32], and these forms would have been excluded.

Our analyses showed significant effects of self-management interventions for respiratory and cardiovascular diseases but failed to indicate clear effects for other long-term conditions. However, over half of the studies included in the review have been conducted among patients with these conditions. Failure to obtain clear results about the effects of self-management interventions on other conditions may partly reflect lack of power.

We only explored basic moderators of effects (such as ‘self-management’ versus ‘case management’). There are a large number of factors on which studies differ, and it is possible in theory to use meta-regression techniques to explore the ‘active ingredients’ of interventions [33, 34]. However, this is dependent on interventions with clearer boundaries and consensus over definitions. The range of self-management interventions included in the current review was very wide. When combined with inconsistent reporting, the utility of meta-regression is more limited. Similarly, the differences in Tables 4 and 5 should be interpreted with caution, as the associations with risk of bias and country of origin may be confounded by other differences between studies.

### Implications of the study for policy and practice

As noted earlier, although many demand management interventions are focussed on high users of health care, many factors (such as the prevalence of high users, as well as artefacts such as regression to the mean) reduce the benefits of intervening in such groups [35]. Self-management support thus has potential to make a large impact on utilisation, because it is relevant to so many patients with long-term conditions, but this assumes that: (a) reductions in utilisation are achieved without compromising other outcomes (b) reductions in utilisation can be consistently achieved (c) self-management support can be disseminated widely.

Our review suggests that very few self-management interventions compromise patient outcomes, at least among those populations consenting to take part in trials. Studies have suggested that self-management *can* lead to such reactions in some patients, particularly those with multimorbidity [36–38], but our data suggests that this is not a consistent outcome.

The core issue thus relates to the impact of self-management support on reducing utilisation. Across conditions, the most robust effects (both in terms of number of studies, and the size of the effects) related to interventions in respiratory and cardiovascular patients, where there was a significant evidence base suggesting consistent (albeit small) reductions in hospital use and costs in both self-management interventions and case management, consistent with other reports in this area [39]. In terms of the wider implementation of self-management support, many trials were based on small, selected samples of volunteer patients, with isolated examples of attempts at more widespread implementation [40, 41].

Understanding the limited and inconsistent impact of self-management requires examination of the assumptions underlying self-management as a demand management strategy. It is assumed that providing patients with self-management support will either lead to indirect utilisation benefits (where self-management leads to better health and thus reduces utilisation) or more direct effects (for example, where self-management enables more effective response to exacerbations and crises, avoiding high cost use such as hospital admission).

There are a number of potential problems with these assumptions. Firstly, self-management interventions will vary in their explicit targeting of utilisation behaviour. As an example, self-management plans to control exacerbations in respiratory disorders often has a core function to avoid unnecessary hospital use. In contrast, self-management in diabetes may have as its aim patient empowerment and the improvement of diabetes control. Variation in impacts between conditions may reflect patterns of service delivery. For example, hospital use related to depression may be relatively rare compared to other conditions. There is also an assumption that utilisation behaviour is patient-led, but some utilisation (such as clinical attendance) may be led by professionals [42]. Health service innovations designed to manage demand may actually create supplier induced demand [35]. Many self-management interventions have fairly limited impacts; health outcomes will set important limits on any indirect effects on utilisation, and even effective self-management interventions may not lead to *enduring* behaviour change. Few studies in the review assessed outcomes over greater than 12 months, and modelling of long term economic consequences of improved health outcomes may be necessary.

### Implications of the study for research

Analyses were hampered by poor reporting of outcome data. We adopted a simple coding of types of self-management interventions, but even assessments of the amount of professional support provided were often difficult. Few studies reported sufficient detail to allow assessment of issues which might impact on replicability of the interventions, especially around the sensitivity of the interventions to particular cultures or health care contexts. More consistent, comprehensive and theory-led reporting of interventions and outcomes would allow much more effective syntheses.

Our analyses suggested that impacts of self-management support were patterned by type of long-term condition, but the utility of disease specific analyses may be limited in the context of increasing focus on multimorbidity. Patients with multiple conditions may face the biggest barriers to self-management [36], but may also have the greatest capacity to benefit [31].

Clearly, further primary research could usefully develop new models of self-management support that could achieve consistent effects on utilisation, following conventional models for the development of complex interventions [43] and drawing on relevant behavioural and social science models relating to patient experience of long-term conditions, as well as those relating to access to care and utilisation.

There is also a need for a broader assessment of the value of self-management in the context of wider service redesign, as many models in this area highlight the interrelationships between patients, professionals, and the wider service context [13, 44], which may be poorly modelled by conventional trials.

## Conclusions

Self-management support interventions rarely compromised patient outcomes. There was evidence that self-management support interventions can reduce hospital use and total costs, although effects were generally small. Evidence for significant reductions in utilisation were strongest for interventions in respiratory and cardiovascular disorders.

Reporting of data relevant to the core research question was poor. Research priorities relate to better reporting of the content of self-management support, exploration of the impact of multimorbidity, and assessment of factors influencing the wider implementation of self-management support.

## Electronic supplementary material

Additional file 1: **Database search strategy for CENTRAL via Cochrane Library (20/06/2012).** (DOCX 957 KB)

Additional file 2: **PRISMA checklist.** (DOC 64 KB)
