# Supplementary material for: Self-management support interventions to reduce health care utilisation without compromising outcomes: a systematic review and meta-analysis
Source: BMC Health Serv Res. 2014 Aug 27;14:356. doi: 10.1186/1472-6963-14-356 (PMC4177163; doi:10.1186/1472-6963-14-356)
Supplement: Supplementary file 1 — Additional file 1: Database search strategy for CENTRAL via Cochrane Library (20/06/2012). (DOCX 957 KB) [file 12913_2014_3495_MOESM1_ESM.docx]

**Database search strategy for CENTRAL via Cochrane Library (20/06/2012)**

| **ID** | **Search** |
| --- | --- |
| #1 | (self NEXT administer*) in Trials |
| #2 | MeSH descriptor Self Administration, this term only |
| #3 | MeSH descriptor Self Care, this term only |
| #4 | "self care" or (selfcare) or (self NEXT manage*) or (selfmonitor*) or (self NEXT monitor*) in Trials |
| #5 | (selfhelp) or "self help" or (self NEXT diagnos*) or (selfdiagnos*) in Trials |
| #6 | (self NEXT assess*) or (selfassess*) in Trials |
| #7 | MeSH descriptor Blood Glucose Self-Monitoring, this term only |
| #8 | "self inititiated intervention" in Trials |
| #9 | (self NEXT initiated NEXT intervent*) in Trials |
| #10 | MeSH descriptor Self Efficacy, this term only |
| #11 | MeSH descriptor Self Medication explode all trees |
| #12 | "self efficacy" or (pharmacist* or pharmacy or pharmacies) NEAR/2 support* in Trials |
| #13 | (pharmacist* or pharmacy or pharmacies) NEAR/2 assist* or (pharmacist* or pharmacy or pharmacies) NEAR/2 (advice or advis* or inform*) or "pharmaceutical care" in Trials |
| #14 | (self NEXT medicat*) or (selfmedicat*) or (self NEXT remed*) or (selfremed*) in Trials |
| #15 | (self NEXT treat*) or (selftreat*) or "self cure" or (selfcure) in Trials |
| #16 | MeSH descriptor Self-Help Groups, this term only |
| #17 | MeSH descriptor Social Support explode all trees |
| #18 | (social NEXT support*) in Trials |
| #19 | (group NEAR/1 (support* or advice or advis* or monitor* or intervention* or train* or instruction or consult* or assist* or education or information)) in Trials |
| #20 | (peer NEAR/1 (support* or advice or advis* or monitor* or intervention* or train* or instruction or consult* or assist* or education or information)) in Trials |
| #21 | (expert NEXT patient*) or "psychosocial support" or (befriend*) or (health NEXT trainer*) in Trials |
| #22 | MeSH descriptor Telemedicine, this term only |
| #23 | (telemedicine) or (telecare) or (telenursing) or (telemonitor*) or (telehealth) in Trials |
| #24 | MeSH descriptor Remote Consultation, this term only |
| #25 | (telephon* or remote or phone) NEAR/2 (follow* or support or consult* or advice or advis* or intervention or train* or instruction or assist* or educate or education or information or monitor*) in Trials |
| #26 | "case management" or (action NEXT plan*) or (management NEXT plan*) or (management NEXT program*) or (care NEXT plan*) in Trials |
| #27 | (nurse NEAR/2 educator*) in Trials |
| #28 | "patient education" in Trials |
| #29 | MeSH descriptor Patient Education as Topic, this term only |
| #30 | MeSH descriptor Case Management, this term only |
| #31 | (patient NEAR/2 (education or advice or advis* or instruct* or educate or train*)) in Trials |
| #32 | "consumer health information" or "patient information" in Trials |
| #33 | (financial or monetary or money) NEAR/2 (incentive* or competition* or contest* or lotter* or reward* or prize*) in Trials |
| #34 | (contingent NEXT payment*) or (deposit NEXT contract*) or (decision NEAR/2 support*) or (decision NEAR/2 aid*) or (shared NEAR/2 decision*) in Trials |
| #35 | MeSH descriptor Decision Making, this term only |
| #36 | (#1 OR #2 OR #3 OR #4 OR #5 OR #6 OR #7 OR #8 OR #9 OR #10 OR #11 OR #12 OR #13 OR #14 OR #15 OR #16 OR #17 OR #18 OR #19 OR #20 OR #21 OR #22 OR #23 OR #24 OR #25 OR #26 OR #27 OR #28 OR #29 OR #30 OR #31 OR #32 OR #33 OR #34 OR #35) |
| #37 | MeSH descriptor Hospitalization explode all trees |
| #38 | MeSH descriptor Health Resources, this term only |
| #39 | (length NEAR/2 stay) or (duration NEAR/2 stay) or (hospital NEAR/1 (visit* or contact* or attendance* or admission* or episode*)) or (time NEAR/2 discharge) or (hospital NEXT day*) in Trials |
| #40 | (patient* or inpatient* or in-patient*) NEAR/1 (cost* or stay) or (number NEAR/2 (nights or days)) in Trials |
| #41 | "primary care" NEAR/2 (visit* or contact* or attendance* or admission* or episode*) or (surgery NEAR/2 (visit* or contact* or attendance* or admission* or episode*)) in Trials |
| #42 | (clinic or surgery or hospital or "accident and emergency") NEAR/2 (work-flow or "work flow") in Trials |
| #43 | (consultation* NEAR/2 (time or length)) or (hospitalization* or hospitalisation* or rehospitalization* or rehospitalisation* or re-hospitalization* or re-hospitalisation*) or "hospital costs" in Trials |
| #44 | (#37 OR #38 OR #39 OR #40 OR #41 OR #42 OR #43) |
| #45 | (#36 AND #44) |
| #46 | MeSH descriptor Economics, this term only |
| #47 | MeSH descriptor Costs and Cost Analysis explode all trees |
| #48 | MeSH descriptor Value of Life, this term only |
| #49 | MeSH descriptor Economics, Dental, this term only |
| #50 | MeSH descriptor Economics, Hospital explode all trees |
| #51 | MeSH descriptor Economics, Medical, this term only |
| #52 | MeSH descriptor Economics, Nursing, this term only |
| #53 | MeSH descriptor Economics, Pharmaceutical, this term only |
| #54 | (#46 OR #47 OR #48 OR #49 OR #50 OR #51 OR #52 OR #53) |
| #55 | econom* or cost or costs or costly or costing or price or prices or pricing or pharmacoeconomic* in Trials |
| #56 | expenditure NOT energy in Trials |
| #57 | value NEAR/2 money in Trials |
| #58 | budget* in Trials |
| #59 | (#55 OR #56 OR #57 OR #58) |
| #60 | (#54 OR #59) |
| #61 | metabolic NEAR/1 cost in Trials |
| #62 | (energy or oxygen) NEAR/1 cost in Trials |
| #63 | (#61 OR #62) |
| #64 | (#60 AND NOT #63) |
| #65 | (#45 AND #60) |

**List of reviews**

1. Aziz NA, Leonardi-Bee J, Phillips M, Gladman JRF, Ledd L, Walker MF: **Therapy-based rehabilitation services for patients living at home more than one year after stroke**. *Cochrane Database of Systematic Reviews* 2008( 2).

2. Bailey JV, Murray E, Rait G, Mercer CH, Morris RW, Peacock R, Cassell J, Nazareth I: **Interactive computer-based interventions for sexual health promotion**. *Cochrane Database of Systematic Reviews* 2010(9).

3. Bola J, Kao D, Soydan H: **Antipsychotic medication for early episode schizophrenia**. *Cochrane Database of Systematic Reviews* 2011(6).

4. Brown JPR, Clark AM, Dalal H, Welch K, Taylor RS: **Patient education in the management of coronary heart disease**. *Cochrane Database of Systematic Reviews* 2011(12).

5. Casas JP, Kwong J, Ebrahim S: **Telemonitoring for chronic heart failure: not ready for prime time.** *Cochrane Database of Systematic Reviews* 2010, **Issue 9. Art. No.: ED000008. DOI: 10.1002/14651858.ED000008.**(9).

6. Currell R, Urquhart C, Wainwright P, Lewis R: **Telemedicine versus face to face patient care: effects on professional practice and health care outcomes**. *The Cochrane database of systematic reviews* 2000(2):CD002098-CD002098.

7. Dale J, Caramlau IO, Lindenmeyer A, Williams SM: **Peer support telephone calls for improving health**. *Cochrane Database of Systematic Reviews* 2008(4).

8. den Boer P, Wiersma D, Russo S, van den Bosch RJ: **Paraprofessionals for anxiety and depressive disorders**. *Cochrane Database of Systematic Reviews* 2005(2).

9. Duke S-AS, Colagiuri S, Colagiuri R: **Individual patient education for people with type 2 diabetes mellitus**. *Cochrane Database of Systematic Reviews* 2009(1).

10. Duncan E, Best C, Hagen S: **Shared decision making interventions for people with mental health conditions**. *Cochrane Database of Systematic Reviews* 2010(1).

11. Durieux P, Trinquart L, Colombet I, Nies J, Walton RT, Rajeswaran A, Walther MR, Harvey E, Burnand B: **Computerized advice on drug dosage to improve prescribing practice**. *Cochrane Database of Systematic Reviews* 2008(3).

12. Ebrahim S, Taylor F, Ward K, Beswick A, Burke M, Smith GD: **Multiple risk factor interventions for primary prevention of coronary heart disease**. *Cochrane Database of Systematic Reviews* 2011(1).

13. Effing T, Monninkhof EM, van der Valk PDLPM, van der Palen J, van Herwaarden CLA, Partidge MR, Walters EH, Zielhuis GA: **Self-management education for patients with chronic obstructive pulmonary disease**. *Cochrane Database of Systematic Reviews* 2007(4).

14. Ellis G, Mant J, Langhorne P, Dennis M, Winner S: **Stroke liaison workers for stroke patients and carers: an individual patient data meta-analysis**. *Cochrane Database of Systematic Reviews* 2010(5).

15. Flodgren G, Deane K, Dickinson HO, Kirk S, Alberti H, Beyer FR, Brown JG, Penney TL, Summerbell CD, Eccles MP: **Interventions to change the behaviour of health professionals and the organisation of care to promote weight reduction in overweight and obese people**. *Cochrane Database of Systematic Reviews* 2010(3).

16. Forster A, Brown L, Smith J, House A, Knapp P, Wright JJ, Young J: **Information provision for stroke patients and their caregivers**. *Cochrane Database of Systematic Reviews* 2012(11).

17. Foster G, Taylor SJC, Eldridge SE, Ramsay J, Griffiths CJ: **Self-management education programmes by lay leaders for people with chronic conditions**. *Cochrane Database of Systematic Reviews* 2007(4).

18. Gibson PG, Coughlan J, Wilson AJ, Abramson M, Bauman A, Hensley MJ, Walters EH: **Self-management education and regular practitioner review for adults with asthma**. *The Cochrane database of systematic reviews* 2000(2):CD001117-CD001117.

19. Gibson PG, Powell H, Coughlan J, Wilson AJ, Abramson M, Haywood P, Bauman A, Hensley MJ, Walters EH: **Self-management education and regular practitioner review for adults with asthma**. *Cochrane database of systematic reviews* 2003(1):CD001117-CD001117.

20. Gibson PG, Powell H, Coughlan J, Wilson AJ, Hensley MJ, Abramson M, Bauman A, Walters EH: **Limited (information only) patient education programs for adults with asthma**. *Cochrane database of systematic reviews* 2002(2):CD001005-CD001005.

21. Gray TA, Orton LC, Henson D, Harper R, Waterman H: **Interventions for improving adherence to ocular hypotensive therapy**. *Cochrane Database of Systematic Reviews* 2009(2).

22. Gross A, Forget M, St George K, Fraser MMH, Graham N, Perry L, Burnie SJ, Goldsmith CH, Haines T, Brunarski D: **Patient education for neck pain**. *Cochrane Database of Systematic Reviews* 2012(3).

23. Haines T, Gross A, Burnie SJ, Goldsmith CH, Perry L: **Patient education for neck pain with or without radiculopathy**. *Cochrane Database of Systematic Reviews* 2009(1).

24. Hall S, Kolliakou A, Petkova H, Froggatt K, Higginson IJ: **Interventions for improving palliative care for older people living in nursing care homes**. *Cochrane Database of Systematic Reviews* 2011(3).

25. Hawthorne K, Robles Y, Cannings-John R, Edwards AGK: **Culturally appropriate health education for type 2 diabetes mellitus in ethnic minority groups**. *Cochrane Database of Systematic Reviews* 2008(3).

26. Heymans MW, van Tulder MW, Esmail R, Bombardier C, Koes BW: **Back schools for non-specific low-back pain**. *The Cochrane database of systematic reviews* 2004(4):CD000261-CD000261.

27. Hoffmann T, Bennett S, Koh C-L, McKenna KT: **Occupational therapy for cognitive impairment in stroke patients**. *Cochrane Database of Systematic Reviews* 2010(9).

28. Kinnersley P, Edwards A, Hood K, Cadbury N, Ryan R, Prout H, Owen D, MacBeth F, Butow P, Butler C: **Interventions before consultations for helping patients address their information needs**. *Cochrane Database of Systematic Reviews* 2007(3).

29. Legg LA, Drummond AE, Langhorne P: **Occupational therapy for patients with problems in activities of daily living after stroke**. *Cochrane Database of Systematic Reviews* 2006(4).

30. Loveman E, Royle P, Waugh N: **Specialist nurses in diabetes mellitus**. *The Cochrane database of systematic reviews* 2003(2):CD003286-CD003286.

31. Malanda UL, Welschen LMC, Ingrid IR, Dekker JM, Nijpels G, Bot SDM: **Self-monitoring of blood glucose in patients with type 2 diabetes mellitus who are not using insulin**. *Cochrane Database of Systematic Reviews* 2012, **Art. No.: CD005060.DOI: 10.1002/14651858.CD005060.pub3**(1).

32. Marshall M, Rathbone J: **Early intervention for psychosis**. *Cochrane Database of Systematic Reviews* 2006(4).

33. Marshall M, Rathbone J: **Early intervention for psychosis**. *Cochrane Database of Systematic Reviews* 2011(6).

34. Martin S, Kelly G, Kernohan WG, McCreight B, Nugent C: **Smart home technologies for health and social care support**. *Cochrane Database of Systematic Reviews* 2008(4).

35. McDonald S, Hetrick S, Green S: **Pre-operative education for hip or knee replacement**. *The Cochrane database of systematic reviews* 2004(1):CD003526-CD003526.

36. McGeough E, Pollock A, Smith LN, Dennis M, Sharpe M, Lewis S, Mead GE: **Interventions for post-stroke fatigue**. *Cochrane Database of Systematic Reviews* 2009(3).

37. McGowan JL, Grad R, Pluye P, Hannes K, Deane K, Labrecque M, Welch V, Tugwell P: **Electronic retrieval of health information by healthcare providers to improve practice and patient care (Review)**. *Cochrane Database of Systematic Reviews* 2009(3).

38. Meader N, Li R, Des Jarlais DC, Pilling S: **Psychosocial interventions for reducing injection and sexual risk behaviour for preventing HIV in drug users**. *Cochrane Database of Systematic Reviews* 2010(1).

39. Montgomery P, Mayo-Wilson E, Dennis J: **Personal assistance for older adults (65+) without dementia**. *Cochrane Database of Systematic Reviews* 2008(1).

40. Morriss RK, Faizal MA, Jones AP, Williamson PR, Bolton C, McCarthy JP: **Interventions for helping people recognise early signs of recurrence in bipolar disorder**. *Cochrane Database of Systematic Reviews* 2007(1).

41. Murray E, Burns J, Tai SS, Lai R, Nazareth I: **Interactive Health Communication Applications for people with chronic disease**. *Cochrane Database of Systematic Reviews* 2005(4).

42. Perkins SJ, Murphy R, Schmidt U, Williams C: **Self-help and guided self-help for eating disorders**. *Cochrane Database of Systematic Reviews* 2006(3).

43. Pharoah F, Mari J, Rathbone J, Wong W: **Family intervention for schizophrenia**. *Cochrane Database of Systematic Reviews* 2010(12).

44. Powell H, Gibson PG: **Options for self-management education for adults with asthma**. *The Cochrane database of systematic reviews* 2003(1):CD004107-CD004107.

45. Reda S, Makhoul S: **Prompts to encourage appointment attendance for people with serious mental illness**. *The Cochrane database of systematic reviews* 2001(2):CD002085-CD002085.

46. Tapp S, Lasserson TJ, Rowe BH: **Education interventions for adults who attend the emergency room for acute asthma**. *Cochrane Database of Systematic Reviews* 2007(3).

47. Taylor S, Bestall J, Cotter S, Falshaw M, Hood S, Parsons S, Wood L, Underwood M: **Clinical service organisation for heart failure - art. no. CD002752.pub2**. *Cochrane Database of Systematic Reviews* 2005(2).

48. Walters JAE, Turnock AC, Walters EH, Wood-Baker R: **Action plans with limited patient education only for exacerbations of chronic obstructive pulmonary disease**. *Cochrane Database of Systematic Reviews* 2010(5).

49. Wetzels R, Harmsen M, Van Weel C, Grol R, Wensing M: **Interventions for improving older patients' involvement in primary care episodes**. *Cochrane Database of Systematic Reviews* 2007(1).

50. Xia J, Merinder LB, Belgamwar MR: **Psychoeducation for schizophrenia**. *Cochrane Database of Systematic Reviews* 2011(6).

51. Young T, Busgeeth K: **Home-based care for reducing morbidity and mortality in people infected with HIV/AIDS**. *Cochrane Database of Systematic Reviews* 2010(1).

52. Casas J-P, Kwong J, Ebrahim S: **Telemonitoring for chronic heart failure: not ready for prime time**. *The Cochrane database of systematic reviews* 2011, **2011**:ED000008-ED000008.

**Studies included in the review**

1. Angermann CE, Stork S, Gelbrich G, Faller H, Jahns R, Frantz S *et al*.: **Mode of action and effects of standardized collaborative disease management on mortality and morbidity in patients with systolic heart failure: the Interdisciplinary Network for Heart Failure (INH) study.** *Circulation: Heart Failure* 2012, **5**.
2. Barnason S, Zimmerman L, Nieveen J, Schulz P, Miller C, Hertzog M *et al*.: **Influence of a symptom management telehealth intervention on older adults' early recovery outcomes after coronary artery bypass surgery.** *Heart & Lung* 2009, **38**.
3. Barton GR, Sach TH, Jenkinson C, Doherty M, Avery AJ, Muir KR: **Lifestyle interventions for knee pain in overweight and obese adults aged > or = 45: economic evaluation of randomised controlled trial.** *BMJ* 2009, **339**.
4. Bauer MS, McBride L, Williford WO, Glick H, Kinosian B, Altshuler L *et al*.: **Collaborative care for bipolar disorder: Part II. Impact on clinical outcome, function, and costs.** *PSYCHIATRIC SERVICES* 2006, **57**.
5. Bauml J, Pitschel-Walz G, Volz A, Engel RR, Kessling W: **Psychoeducation in schizophrenia: 7-year follow-up concerning rehospitalization and days in hospital in the Munich Psychosis Information Project Study.** *JOURNAL OF CLINICAL PSYCHIATRY* 2007, **68**.
6. Beck A, Scott J, Williams P, Robertson B, Jackson D, Gade G *et al*.: **A randomized trial of group outpatient visits for chronically ill older HMO members: the Cooperative Health Care Clinic.** *Journal of the American Geriatrics Society* 1997, **45**.
7. Beckerman M, Magadle R, Weiner M, Weiner P: **The effects of 1 year of specific inspiratory muscle training in patients with COPD.** *CHEST* 2005, **128**.
8. Behnke M, Jorres RA, Kirsten D, Magnussen H: **Clinical benefits of a combined hospital and home-based exercise programme over 18 months in patients with severe COPD.** *MONALDI ARCHIVES FOR CHEST DISEASE* 2003, **59**.
9. Bocchi EA, Cruz F, Guimaraes G, Pinho Moreira LF, Issa VS, Ayub Ferreira SM *et al*.: **Long-term prospective, randomized, controlled study using repetitive education at six-month intervals and monitoring for adherence in heart failure outpatients: the REMADHE trial.** *Circulation: Heart Failure* 2008, **1**.
10. Bosmans J, de Bruijne M, van Hout H, van Marwijk H, Beekman A, Bouter L *et al*.: **Cost-effectiveness of a disease management program for major depression in elderly primary care patients.** *JOURNAL OF GENERAL INTERNAL MEDICINE* 2006, **21**.
11. Bosmans JE, Brook OH, van Hout HPJ, de Bruijne MC, Nieuwenhuyse H, Bouter LM *et al*.: **Cost effectiveness of a pharmacy-based coaching programme to improve adherence to antidepressants.** *PHARMACOECONOMICS* 2007, **25**.
12. Bouvy ML, Heerdink ER, Urquhart J, Grobbee DE, Hoes AW, Leufkens HGM: **Effect of a pharmacist-led intervention on diuretic compliance in heart failure patients: a randomized controlled study.[Erratum appears in J Card Fail. 2003 Dec;9(6):481 Note: Hoe Arno W [corrected to Hoes Arno W]].** *JOURNAL OF CARDIAC FAILURE* 2003, **9**.
13. Boxall A-M, Barclay L, Sayers A, Caplan GA: **Managing chronic obstructive pulmonary disease in the community. A randomized controlled trial of home-based pulmonary rehabilitation for elderly housebound patients.** *JOURNAL OF CARDIOPULMONARY REHABILITATION* 2005, **25**.
14. Brotons C, Falces C, Alegre J, Ballarin E, Casanovas J, Cata T *et al*.: **Randomized clinical trial of the effectiveness of a home-based intervention in patients with heart failure: the IC-DOM study.** *REVISTA ESPANOLA DE CARDIOLOGIA* 2009, **62**.
15. Brun JF, Bordenave S, Mercier J, Jaussent A, Picot MC, Prefaut C: **Cost-sparing effect of twice-weekly targeted endurance training in type 2 diabetics: a one-year controlled randomized trial.** *Diabetes & Metabolism* 2008, **34**.
16. Bulthuis Y, Mohammad S, Braakman-Jansen LM, Drossaers-Bakker KW, van de Laar MA: **Cost-effectiveness of intensive exercise therapy directly following hospital discharge in patients with arthritis: results of a randomized controlled clinical trial.** *Arthritis Rheum* 2008, **59**.
17. Capomolla S, Febo O, Ceresa M, Caporotondi A, Guazzotti G, La Rovere M *et al*.: **Cost/utility ratio in chronic heart failure: comparison between heart failure management program delivered by day-hospital and usual care.** *JOURNAL OF THE AMERICAN COLLEGE OF CARDIOLOGY* 2002, **40**.
18. Castro M, Zimmermann NA, Crocker S, Bradley J, Leven C, Schechtman KB: **Asthma intervention program prevents readmissions in high healthcare users.** *AMERICAN JOURNAL OF RESPIRATORY & CRITICAL CARE MEDICINE* 2003, **168**.
19. Clark NM, Gong ZM, Wang SJ, Lin X, Bria WF, Johnson TR: **A randomized trial of a self-regulation intervention for women with asthma.** *Chest* 2007, **132**.
20. Clarke G, Eubanks D, Reid E, Kelleher C, O'Connor E, DeBar LL *et al*.: **Overcoming Depression on the Internet (ODIN) (2): a randomized trial of a self-help depression skills program with reminders.** *Journal of Medical Internet Research* 2005, **7**.
21. Cline CM, Israelsson BY, Willenheimer RB, Broms K, Erhardt LR: **Cost effective management programme for heart failure reduces hospitalisation.** *HEART* 1998, **80**.
22. Community Pharmacy Medicines Management Project Evaluation Team: **The MEDMAN study: a randomized controlled trial of community pharmacy-led medicines management for patients with coronary heart disease.** *FAMILY PRACTICE* 2007, **24**.
23. Coull AJ, Taylor VH, Elton R, Murdoch PS, Hargreaves AD: **A randomised controlled trial of senior Lay Health Mentoring in older people with ischaemic heart disease: The Braveheart Project.** *Age & Ageing* 2004, **33**.
24. Coultas D, Frederick J, Barnett B, Singh G, Wludyka P: **A randomized trial of two types of nurse-assisted home care for patients with COPD.** *CHEST* 2005, **128**.
25. Davidson PM, Cockburn J, Newton PJ, Webster JK, Betihavas V, Howes L *et al*.: **Can a heart failure-specific cardiac rehabilitation program decrease hospitalizations and improve outcomes in high-risk patients?** *European journal of cardiovascular prevention & rehabilitation* 2010, **17**.
26. Davies M, Dixon S, Currie CJ, Davis RE, Peters JR: **Evaluation of a hospital diabetes specialist nursing service: a randomized controlled trial.** *DIABETIC MEDICINE* 2001, **18**.
27. de la Porte PWFB-A, Lok DJA, van Veldhuisen DJ, van Wijngaarden J, Cornel JH, Zuithoff NPA *et al*.: **Added value of a physician-and-nurse-directed heart failure clinic: results from the Deventer-Alkmaar heart failure study.** *HEART* 2007, **93**.
28. Dekker RL, Moser DK, Peden AR, Lennie TA: **Cognitive therapy improves three-month outcomes in hospitalized patients with heart failure.** *JOURNAL OF CARDIAC FAILURE* 2012, **18**.
29. den Boer PCAM, Wiersma D, Ten Vaarwerk I, Span MM, Stant AD, Van den Bosch RJ: **Cognitive self-therapy for chronic depression and anxiety: a multi-centre randomized controlled study.** *Psychological medicine* 2007, **37**.
30. DeWalt DA, Baker DW, Schillinger D, Hawk V, Ruo B, Bibbins-Domingo K *et al*.: **A Multisite randomized trial of a single-versus multi-session literacy sensitive self-care intervention for patients with heart failure.** *Journal of General Internal Medicine Conference: 34th Annual Meeting of the Society of General Internal Medicine Phoenix, AZ United States Conference Start* 2011, **26**.
31. DeWalt DA, Malone RM, Bryant ME, Kosnar MC, Corr KE, Rothman RL *et al*.: **A heart failure self-management program for patients of all literacy levels: a randomized, controlled trial [ISRCTN11535170].** *BMC HEALTH SERVICES RESEARCH* 2006, **6**.
32. Doughty RN, Wright SP, Pearl A, Walsh HJ, Muncaster S, Whalley GA *et al*.: **Randomized, controlled trial of integrated heart failure management: The Auckland Heart Failure Management Study.** *EUROPEAN HEART JOURNAL* 2002, **23**.
33. Druss BG, Rohrbaugh RM, Levinson CM, Rosenheck RA: **Integrated medical care for patients with serious psychiatric illness: a randomized trial.** *Archives of general psychiatry* 2010, **58**.
34. Dunagan WC, Littenberg B, Ewald GA, Jones CA, Emery VB, Waterman BM *et al*.: **Randomized trial of a nurse-administered, telephone-based disease management program for patients with heart failure.** *JOURNAL OF CARDIAC FAILURE* 2005, **11**.
35. Dunbar SB, Langberg JJ, Reilly CM, Viswanathan B, McCarty F, Culler SD *et al*.: **Effect of a psychoeducational intervention on depression, anxiety, and health resource use in implantable cardioverter defibrillator patients.** *Pacing & Clinical Electrophysiology* 2009, **32**.
36. Dunn NJ, Rehm LP, Schillaci J, Souchek J, Mehta P, Ashton CM *et al*.: **A randomized trial of self-management and psychoeducational group therapies for comorbid chronic posttraumatic stress disorder and depressive disorder.** *JOURNAL OF TRAUMATIC STRESS* 2007, **20**.
37. Eaton T, Young P, Fergusson W, Moodie L, Zeng I, O'KANE F *et al*.: **Does early pulmonary rehabilitation reduce acute health-care utilization in COPD patients admitted with an exacerbation? A randomized controlled study.** *Respirology* 2009, **14**.
38. Gallefoss F, Bakke PS: **Cost-effectiveness of self-management in asthmatics: a 1-yr follow-up randomized, controlled trial.** *EUROPEAN RESPIRATORY JOURNAL* 2001, **17**.
39. Gesica Investigators: **Randomised trial of telephone intervention in chronic heart failure: DIAL trial.** *BMJ* 2005, **331**.
40. Gillett M, Dallosso HM, Dixon S, Brennan A, Carey ME, Campbell MJ *et al*.: **Delivering the diabetes education and self management for ongoing and newly diagnosed (DESMOND) programme for people with newly diagnosed type 2 diabetes: cost effectiveness analysis.** *BMJ* 2010, **341**.
41. Goldberg LR, Piette JD, Walsh MN, Frank TA, Jaski BE, Smith AL *et al*.: **Randomized trial of a daily electronic home monitoring system in patients with advanced heart failure: the Weight Monitoring in Heart Failure (WHARF) trial.** *American heart journal* 2003, **146**.
42. Griffiths C, Motlib J, Azad A, Ramsay J, Eldridge S, Feder G *et al*.: **Randomised controlled trial of a lay-led self-management programme for Bangladeshi patients with chronic disease.** *BRITISH JOURNAL OF GENERAL PRACTICE* 2005, **55**.
43. Groessl EJ, Cronan TA: **A cost analysis of self-management programs for people with chronic illness.** *AMERICAN JOURNAL OF COMMUNITY PSYCHOLOGY* 2000, **28**.
44. Gruffydd-Jones K, Hollinghurst S, Ward S, Taylor G: **Targeted routine asthma care in general practice using telephone triage.** *BRITISH JOURNAL OF GENERAL PRACTICE* 2005, **55**.
45. Guell R, Casan P, Belda J, Sangenis M, Morante F, Guyatt GH *et al*.: **Long-term effects of outpatient rehabilitation of COPD: A randomized trial.** *Chest* 2000, **117**.
46. Haas M, Groupp E, Muench J, Kraemer D, Brummel-Smith K, Sharma R *et al*.: **Chronic disease self-management program for low back pain in the elderly.** *J Manipulative Physiol Ther* 2005, **28**.
47. Handley MA, Shumway M, Schillinger D: **Cost-effectiveness of automated telephone self-management support with nurse care management among patients with diabetes.** *ANNALS OF FAMILY MEDICINE* 2008, **6**.
48. Hanssen TA, Nordrehaug JE, Eide GE, Hanestad BR: **Does a telephone follow-up intervention for patients discharged with acute myocardial infarction have long-term effects on health-related quality of life? A randomised controlled trial.** *Journal of Clinical Nursing* 2009, **18**.
49. Henderson C, Knapp M, Fernandez JL, Beecham J, Hirani SP, Cartwright M *et al*.: **Cost effectiveness of telehealth for patients with long term conditions (Whole Systems Demonstrator telehealth questionnaire study): nested economic evaluation in a pragmatic, cluster randomised controlled trial.** *BMJ* 2013, **346**.
50. Hermiz O, Comino E, Marks G, Daffurn K, Wilson S, Harris M: **Randomised controlled trial of home based care of patients with chronic obstructive pulmonary disease.** *BMJ* 2002, **325**.
51. Hernandez C, Casas A, Escarrabill J, Alonso J, Puig-Junoy J, Farrero E *et al*.: **Home hospitalisation of exacerbated chronic obstructive pulmonary disease patients.** *EUROPEAN RESPIRATORY JOURNAL* 2003, **21**.
52. Holland R, Brooksby I, Lenaghan E, Ashton K, Hay L, Smith R *et al*.: **Effectiveness of visits from community pharmacists for patients with heart failure: HeartMed randomised controlled trial.** *BMJ* 2007, **334**.
53. Hurley MV, Walsh NE, Mitchell HL, Pimm TJ, Patel A, Williamson E *et al*.: **Clinical effectiveness of a rehabilitation program integrating exercise, self-management, and active coping strategies for chronic knee pain: a cluster randomized trial.** *ARTHRITIS & RHEUMATISM* 2007, **57**.
54. Hurley MV, Walsh NE, Mitchell HL, Pimm TJ, Williamson E, Jones RH *et al*.: **Economic evaluation of a rehabilitation program integrating exercise, self-management, and active coping strategies for chronic knee pain.** *ARTHRITIS & RHEUMATISM* 2007, **57**.
55. Irvine L, Barton GR, Gasper AV, Murray N, Clark A, Scarpello T *et al*.: **Cost-effectiveness of a lifestyle intervention in preventing Type 2 diabetes.** *INTERNATIONAL JOURNAL OF TECHNOLOGY ASSESSMENT IN HEALTH CARE* 2011, **27**.
56. Jansa M, Vidal M, Viaplana J, Levy I, Conget I, Gomis R *et al*.: **Telecare in a structured therapeutic education programme addressed to patients with type 1 diabetes and poor metabolic control.** *Diabetes Research & Clinical Practice* 2006, **74**.
57. Jayadevappa R, Johnson JC, Bloom BS, Nidich S, Desai S, Chhatre S *et al*.: **Effectiveness of transcendental meditation on functional capacity and quality of life of African Americans with congestive heart failure: a randomized control study.[Erratum appears in Ethn Dis. 2007 Summer;17(3):595], [Summary for patients in Ethn Dis. 2007 Winter;17(1):172-3; PMID: 17278286].** *Ethnicity & Disease* 2007, **17**.
58. Jerant A, Moore-Hill M, Franks P: **Home-based, peer-led chronic illness self-management training: findings from a 1-year randomized controlled trial.** *ANNALS OF FAMILY MEDICINE* 2009, **7**.
59. Jessep SA, Walsh NE, Ratcliffe J, Hurley MV: **Long-term clinical benefits and costs of an integrated rehabilitation programme compared with outpatient physiotherapy for chronic knee pain.** *PHYSIOTHERAPY* 2009, **95**.
60. Johnson RE, Jones GT, Wiles NJ, Chaddock C, Potter RG, Roberts C *et al*.: **Active exercise, education, and cognitive behavioral therapy for persistent disabling low back pain: a randomized controlled trial.** *SPINE* 2007, **32**.
61. Jolly K, Bradley F, Sharp S, Smith H, Thompson S, Kinmonth AL *et al*.: **Randomised controlled trial of follow up care in general practice of patients with myocardial infarction and angina: final results of the Southampton heart integrated care project (SHIP). The SHIP Collaborative Group.** *BMJ* 1999, **318**.
62. Jolly K, Taylor R, Lip GYH, Greenfield S, Raftery J, Mant J *et al*.: **The Birmingham Rehabilitation Uptake Maximisation Study (BRUM). Home-based compared with hospital-based cardiac rehabilitation in a multi-ethnic population: cost-effectiveness and patient adherence.** *Health Technology Assessment* 2007, **11**.
63. Karjalainen K, Malmivaara A, Pohjolainen T, Hurri H, Mutanen P, Rissanen P *et al*.: **Mini-intervention for subacute low back pain: a randomized controlled trial.** *SPINE* 2003, **28**.
64. Kasper EK, Gerstenblith G, Hefter G, Van Anden E, Brinker JA, Thiemann DR *et al*.: **A randomized trial of the efficacy of multidisciplinary care in heart failure outpatients at high risk of hospital readmission.** *JOURNAL OF THE AMERICAN COLLEGE OF CARDIOLOGY* 2002, **39**.
65. Katon W, Russo J, Sherbourne C, Stein MB, Craske M, Fan MY *et al*.: **Incremental cost-effectiveness of a collaborative care intervention for panic disorder.** *Psychol Med* 2006, **36**.
66. Katon W, Russo J, Von Korff M, Lin E, Simon G, Bush T *et al*.: **Long-term effects of a collaborative care intervention in persistently depressed primary care patients.** *JOURNAL OF GENERAL INTERNAL MEDICINE* 2002, **17**.
67. Katon WJ, Lin EHB, Von Korff M, Ciechanowski P, Ludman EJ, Young B *et al*.: **Collaborative care for patients with depression and chronic illnesses.** *NEW ENGLAND JOURNAL OF MEDICINE* 2010, **363**.
68. Katon WJ, Roy-Byrne P, Russo J, Cowley D: **Cost-effectiveness and cost offset of a collaborative care intervention for primary care patients with panic disorder.** *Archives of general psychiatry* 2002, **59**.
69. Katon WJ, Schoenbaum M, Fan M-Y, Callahan CM, Williams J, Jr., Hunkeler E *et al*.: **Cost-effectiveness of improving primary care treatment of late-life depression.** *Archives of general psychiatry* 2005, **62**.
70. Kauppinen R, Sintonen H, Tukiainen H: **One-year economic evaluation of intensive vs conventional patient education and supervision for self-management of new asthmatic patients.** *RESPIRATORY MEDICINE* 1998, **92**.
71. Kennedy A, Reeves D, Bower P, Lee V, Middleton E, Richardson G *et al*.: **The effectiveness and cost effectiveness of a national lay-led self care support programme for patients with long-term conditions: a pragmatic randomised controlled trial.** *Journal of Epidemiology & Community Health* 2007, **61**.
72. Khdour MR, Agus AM, Kidney JC, Smyth BM, Elnay JC, Crealey GE: **Cost-utility analysis of a pharmacy-led self-management programme for patients with COPD.** *International Journal of Clinical Pharmacy* 2011, **33**.
73. Ko FW, David DL, Ngai JC, Tung A, Ng S, Lau H *et al*.: **Effect of Early Pulmonary Rehabilitation on Health-care Utilization and Health Status in Patients Hospitalized With Acute Exacerbations of COPD.** *CHEST Journal* 2010, **138**.
74. Koehler F, Winkler S, Schieber M, Sechtem U, Stangl K, Bohm M *et al*.: **Impact of remote telemedical management on mortality and hospitalizations in ambulatory patients with chronic heart failure: the telemedical interventional monitoring in heart failure study.** *CIRCULATION* 2011, **123**.
75. Koff PB, Jones RH, Cashman JM, Voelkel NF, Vandivier RW: **Proactive integrated care improves quality of life in patients with COPD.** *EUROPEAN RESPIRATORY JOURNAL* 2009, **33**.
76. Kroenke K, Theobald D, Wu J, Loza JK, Carpenter JS, Tu W: **The association of depression and pain with health-related quality of life, disability, and health care use in cancer patients.** *Journal of Pain & Symptom Management* 2010, **40**.
77. Kwok T, Lee J, Woo J, Lee DT, Griffith S: **A randomized controlled trial of a community nurse-supported hospital discharge programme in older patients with chronic heart failure.** *Journal of Clinical Nursing* 2008, **17**.
78. Lahdensuo A, Haahtela T, Herrala J, Kava T, Kiviranta K, Kuusisto P *et al*.: **Randomised comparison of guided self management and traditional treatment of asthma over one year.** *BMJ* 1996, **312**.
79. Lee DT, Lee IF, Mackenzie AE, Ho RN: **Effects of a care protocol on care outcomes in older nursing home patients with chronic obstructive pulmonary disease.** *J Am Geriatr Soc* 2002, **50**.
80. Levitt AJ, Mueser KT, Degenova J, Lorenzo J, Bradford-Watt D, Barbosa A *et al*.: **Randomized controlled trial of illness management and recovery in multiple-unit supportive housing.** *PSYCHIATRIC SERVICES* 2009, **60**.
81. Levy ML, Robb M, Allen J, Doherty C, Bland JM, Winter RJ: **A randomized controlled evaluation of specialist nurse education following accident and emergency department attendance for acute asthma.** *RESPIRATORY MEDICINE* 2000, **94**.
82. Lewin B, Robertson IH, Cay EL, Irving JB, Campbell M: **Effects of self-help post-myocardial-infarction rehabilitation on psychological adjustment and use of health services.** *LANCET* 1992, **339**.
83. Lewin RJ, Coulton S, Frizelle DJ, Kaye G, Cox H: **A brief cognitive behavioural preimplantation and rehabilitation programme for patients receiving an implantable cardioverter-defibrillator improves physical health and reduces psychological morbidity and unplanned readmissions.** *HEART* 2009, **95**.
84. Linton SJ, Andersson T: **Can chronic disability be prevented? A randomized trial of a cognitive-behavior intervention and two forms of information for patients with spinal pain.** *Spine* 2000, **25**.
85. Lopez Cabezas C, Falces Salvador C, Cubi Quadrada D, Arnau Bartes A, Ylla Bore M, Muro Perea N *et al*.: **Randomized clinical trial of a postdischarge pharmaceutical care program vs regular follow-up in patients with heart failure.** *Farmacia Hospitalaria* 2006, **30**.
86. Man WD, Polkey MI, Donaldson N, Gray BJ, Moxham J: **Community pulmonary rehabilitation after hospitalisation for acute exacerbations of chronic obstructive pulmonary disease: randomised controlled study.** *BMJ: British Medical Journal* 2004, **329**.
87. Mancuso CA, Peterson MGE, Gaeta TJ, Fernandez JL, Birkhahn RH, Melniker LA *et al*.: **A randomized controlled trial of self-management education for asthma patients in the emergency department.** *ANNALS OF EMERGENCY MEDICINE* 2011, **57**.
88. Markle-Reid M, Orridge C, Weir R, Browne G, Gafni A, Lewis M *et al*.: **Interprofessional stroke rehabilitation for stroke survivors using home care.** *CANADIAN JOURNAL OF NEUROLOGICAL SCIENCES* 2011, **38**.
89. McBeth J, Prescott G, Scotland G, Lovell K, Keeley P, Hannaford P *et al*.: **Cognitive behavior therapy, exercise, or both for treating chronic widespread pain.** *ARCHIVES OF INTERNAL MEDICINE* 2012, **172**.
90. McDonald K, Ledwidge M, Cahill J, Quigley P, Maurer B, Travers B *et al*.: **Heart failure management: multidisciplinary care has intrinsic benefit above the optimization of medical care.** *Journal of Cardiac Failure* 2002, **8**.
91. McGeoch GRB, Willsman KJ, Dowson CA, Town GI, Frampton CM, McCartin FJ *et al*.: **Self-management plans in the primary care of patients with chronic obstructive pulmonary disease.** *Respirology* 2006, **11**.
92. McGowan P: **The efficacy of diabetes patient education and self-management education in type 2 diabetes.** *Canadian Journal of Diabetes* 2011, **35**.
93. McLean W, Gillis J, Waller R: **The BC Community Pharmacy Asthma Study: A study of clinical, economic and holistic outcomes influenced by an asthma care protocol provided by specially trained community pharmacists in British Columbia.** *Canadian Respiratory Journal* 2003, **10**.
94. McWilliam CL, Stewart M, Brown JB, McNair S, Donner A, Desai K *et al*.: **Home-based health promotion for chronically ill older persons: results of a randomized controlled trialof a critical reflection approach.** *Health promotion international* 1999, **14**.
95. Meijer EM, Sluiter JK, Heyma A, Sadiraj K, Frings-Dresen MH: **Cost-effectiveness of multidisciplinary treatment in sick-listed patients with upper extremity musculoskeletal disorders: a randomized, controlled trial with one-year follow-up.** *Int Arch Occup Environ Health* 2006, **79**.
96. Mejhert M, Kahan T, Persson H, Edner M: **Limited long term effects of a management programme for heart failure.** *HEART* 2004, **90**.
97. Moffett JK, Torgerson D, Bell-Syer S, Jackson D, Llewlyn-Phillips H, Farrin A *et al*.: **Randomised controlled trial of exercise for low back pain: clinical outcomes, costs, and preferences.** *BMJ* 1999, **319**.
98. Monninkhof E, van der Valk P, Schermer T, van der Palen J, van Herwaarden C, Zielhuis G: **Economic evaluation of a comprehensive self-management programme in patients with moderate to severe chronic obstructive pulmonary disease.** *Chronic Respiratory Disease* 2004, **1**.
99. Morcillo C, Valderas JM, Aguado O, Delas J, Sort D, Pujadas R *et al*.: **[Evaluation of a home-based intervention in heart failure patients. Results of a randomized study].** *REVISTA ESPANOLA DE CARDIOLOGIA* 2005, **58**.
100. Moudgil H, Marshall T, Honeybourne D: **Asthma education and quality of life in the community: a randomised controlled study to evaluate the impact on white European and Indian subcontinent ethnic groups from socioeconomically deprived areas in Birmingham, UK.** *Thorax* 2000, **55**.
101. Murphy AW, Cupples ME, Smith SM, Byrne M, Byrne MC, Newell J *et al*.: **Effect of tailored practice and patient care plans on secondary prevention of heart disease in general practice: cluster randomised controlled trial.** *BMJ* 2009, **339**.
102. Murray MD, Young J, Hoke S, Tu W, Weiner M, Morrow D *et al*.: **Pharmacist intervention to improve medication adherence in heart failure: a randomized trial.** *ANNALS OF INTERNAL MEDICINE* 2007, **146**.
103. Naylor MD, Brooten DA, Campbell RL, Maislin G, McCauley KM, Schwartz JS: **Transitional care of older adults hospitalized with heart failure: a randomized, controlled trial.[Erratum appears in J Am Geriatr Soc. 2004 Jul;52(7):1228].** *Journal of the American Geriatrics Society* 2004, **52**.
104. Niemisto L, Lahtinen-Suopanki T, Rissanen P, Lindgren K-A, Sarna S, Hurri H: **A randomized trial of combined manipulation, stabilizing exercises, and physician consultation compared to physician consultation alone for chronic low back pain.** *SPINE* 2003, **28**.
105. Ninot G, Moullec G, Picot MC, Jaussent A, Hayot M, Desplan M *et al*.: **Cost-saving effect of supervised exercise associated to COPD self-management education program.** *RESPIRATORY MEDICINE* 2011, **105**.
106. Nucifora G, Albanese MC, De Biaggio P, Caliandro D, Gregori D, Goss P *et al*.: **Lack of improvement of clinical outcomes by a low-cost, hospital-based heart failure management programme.** *Journal of Cardiovascular Medicine* 2006, **7**.
107. Nunez M, Nunez E, Segur JM, Macule F, Quinto L, Hernandez MV *et al*.: **The effect of an educational program to improve health-related quality of life in patients with osteoarthritis on waiting list for total knee replacement: a randomized study.** *Osteoarthritis Cartilage* 2006, **14**.
108. Ojeda S, Anguita M, Delgado M, Atienza F, Rus C, Granados AL *et al*.: **Short- and long-term results of a programme for the prevention of readmissions and mortality in patients with heart failure: are effects maintained after stopping the programme?** *European Journal of Heart Failure* 2005, **7**.
109. Patel A, Buszewicz M, Beecham J, Griffin M, Rait G, Nazareth I *et al*.: **Economic evaluation of arthritis self management in primary care.** *BMJ* 2009, **339**.
110. Penn DL, Meyer PS, Evans E, Wirth RJ, Cai K, Burchinal M: **A randomized controlled trial of group cognitive-behavioral therapy vs. enhanced supportive therapy for auditory hallucinations.** *SCHIZOPHRENIA RESEARCH* 2009, **109**.
111. Penn DL, Uzenoff SR, Perkins D, Mueser KT, Hamer R, Waldheter E *et al*.: **A pilot investigation of the Graduated Recovery Intervention Program (GRIP) for first episode psychosis.** *SCHIZOPHRENIA RESEARCH* 2011, **125**.
112. Peters J, Large RG, Elkind G: **Follow-up results from a randomised controlled trial evaluating in- and outpatient pain management programmes.** *PAIN* 1992, **50**.
113. Peters JL, Large RG: **A randomised control trial evaluating in- and outpatient pain management programmes.** *PAIN* 1990, **41**.
114. Pilotto LS, Smith BJ, Heard AR, McElroy HJ, Weekley J, Bennett P: **Trial of nurse-run asthma clinics based in general practice versus usual medical care.** *Respirology* 2004, **9**.
115. Price D, Haughney J, Lloyd A, Hutchinson J, Plumb J: **An economic evaluation of adjustable and fixed dosing with budesonide/formoterol via a single inhaler in asthma patients: the ASSURE study.** *Curr Med Res Opin* 2004, **20**.
116. Pyne JM, Fortney JC, Tripathi SP, Maciejewski ML, Edlund MJ, Williams DK: **Cost-effectiveness analysis of a rural telemedicine collaborative care intervention for depression.** *Archives of general psychiatry* 2010, **67**.
117. Ramachandran K, Husain N, Maikhuri R, Seth S, Vij A, Kumar M *et al*.: **Impact of a comprehensive telephone-based disease management programme on quality-of-life in patients with heart failure.** *The National medical journal of India* 2007, **20**.
118. Rea H, McAuley S, Stewart A, Lamont C, Roseman P, Didsbury P: **A chronic disease management programme can reduce days in hospital for patients with chronic obstructive pulmonary disease.** *INTERNAL MEDICINE JOURNAL* 2004, **34**.
119. Reynolds W, Lauder W, Sharkey S, Maciver S, Veitch T, Cameron D: **The effects of a transitional discharge model for psychiatric patients.** *Journal of Psychiatric & Mental Health Nursing* 2004, **11**.
120. Rich MW, Beckham V, Wittenberg C, Leven CL, Freedland KE, Carney RM: **A multidisciplinary intervention to prevent the readmission of elderly patients with congestive heart failure.** *NEW ENGLAND JOURNAL OF MEDICINE* 1995, **333**.
121. Richardson G, Sculpher M, Kennedy A, Nelson E, Reeves D, Roberts C *et al*.: **Is self-care a cost-effective use of resources? Evidence from a randomized trial in inflammatory bowel disease.** *Journal of Health Services & Research Policy* 2006, **11**.
122. Richardson J, Letts L, Chan D, Stratford P, Hand C, Price D *et al*.: **Rehabilitation in a primary care setting for persons with chronic illness--A randomized controlled trial.** *Primary Health Care Research and Development* 2010, **11**.
123. Riegel B, Carlson B, Glaser D, Romero T: **Randomized controlled trial of telephone case management in Hispanics of Mexican origin with heart failure.** *JOURNAL OF CARDIAC FAILURE* 2006, **12**.
124. Ries AL, Kaplan RM, Myers R, Prewitt LM: **Maintenance after pulmonary rehabilitation in chronic lung disease: a randomized trial.** *Am J Respir Crit Care Med* 2003, **167**.
125. Rivera JJ, Sullivan AM, Valenti SS: **Adding consumer-providers to intensive case management: does it improve outcome?** *PSYCHIATRIC SERVICES* 2007, **58**.
126. Roberts J, Browne GB, Streiner D, Gafni A, Pallister R, Hoxby H *et al*.: **The effectiveness and efficiency of health promotion in specialty clinic care.** *MEDICAL CARE* 1995, **33**.
127. Roelofs PDDM, Bierma-Zeinstra SMA, van Poppel MNM, van Mechelen W, Koes BW, van Tulder MW: **Cost-effectiveness of lumbar supports for home care workers with recurrent low back pain: an economic evaluation alongside a randomized-controlled trial.** *SPINE* 2010, **35**.
128. Ryan D, Price D, Musgrave SD, Malhotra S, Lee AJ, Ayansina D *et al*.: **Clinical and cost effectiveness of mobile phone supported self monitoring of asthma: multicentre randomised controlled trial.** *BMJ* 2012, **344**.
129. Schermer TR, Thoonen BP, van den Boom G, Akkermans RP, Grol RP, Folgering HT *et al*.: **Randomized controlled economic evaluation of asthma self-management in primary health care.** *AMERICAN JOURNAL OF RESPIRATORY & CRITICAL CARE MEDICINE* 2002, **166**.
130. Schwarz KA, Mion LC, Hudock D, Litman G: **Telemonitoring of heart failure patients and their caregivers: a pilot randomized controlled trial.** *PROGRESS IN CARDIOVASCULAR NURSING* 2008, **23**.
131. Seto E, Leonard KJ, Cafazzo JA, Barnsley J, Masino C, Ross HJ: **Mobile phone-based telemonitoring for heart failure management: a randomized controlled trial.** *Journal of Medical Internet Research* 2012, **14**.
132. Seymour JM, Moore L, Jolley CJ, Ward K, Creasey J, Steier JS *et al*.: **Outpatient pulmonary rehabilitation following acute exacerbations of COPD.** *Thorax* 2010, **65**.
133. Shelledy DC, Legrand TS, Gardner DD, Peters JI: **A randomized, controlled study to evaluate the role of an in-home asthma disease management program provided by respiratory therapists in improving outcomes and reducing the cost of care.** *JOURNAL OF ASTHMA* 2009, **46**.
134. Simon GE, Katon WJ, VonKorff M, Unutzer J, Lin EH, Walker EA *et al*.: **Cost-effectiveness of a collaborative care program for primary care patients with persistent depression.** *AMERICAN JOURNAL OF PSYCHIATRY* 2001, **158**.
135. Simon GE, Ludman EJ, Bauer MS, Unutzer J, Operskalski B: **Long-term effectiveness and cost of a systematic care program for bipolar disorder.** *Archives of general psychiatry* 2006, **63**.
136. Simon GE, Ludman EJ, Rutter CM: **Incremental benefit and cost of telephone care management and telephone psychotherapy for depression in primary care.** *Archives of general psychiatry* 2009, **66**.
137. Simon GE, Von Korff M, Ludman EJ, Katon WJ, Rutter C, Unutzer J *et al*.: **Cost-effectiveness of a program to prevent depression relapse in primary care.** *MEDICAL CARE* 2002, **40**.
138. Simon J, Gray A, Clarke P, Wade A, Neil A, Farmer A *et al*.: **Cost effectiveness of self monitoring of blood glucose in patients with non-insulin treated type 2 diabetes: economic evaluation of data from the DiGEM trial.** *BMJ* 2008, **336**.
139. Sinclair AJ, Conroy SP, Davies M, Bayer AJ: **Post-discharge home-based support for older cardiac patients: a randomised controlled trial.** *Age & Ageing* 2005, **34**.
140. Sisk JE, Hebert PL, Horowitz CR, McLaughlin MA, Wang JJ, Chassin MR: **Effects of nurse management on the quality of heart failure care in minority communities: a randomized trial.[Summary for patients in Ann Intern Med. 2006 Aug 15;145(4):I28; PMID: 16908913].** *ANNALS OF INTERNAL MEDICINE* 2006, **145**.
141. Soler JJ, Martinez-Garcia MA, Roman P, Orero R, Terrazas S, Martinez-Pechuan A: **[Effectiveness of a specific program for patients with chronic obstructive pulmonary disease and frequent exacerbations].** *ARCHIVOS DE BRONCONEUMOLOGIA* 2006, **42**.
142. Solomon DH, Warsi A, Brown-Stevenson T, Farrell M, Gauthier S, Mikels D *et al*.: **Does self-management education benefit all populations with arthritis? A randomized controlled trial in a primary care physician network.** *JOURNAL OF RHEUMATOLOGY* 2002, **29**.
143. Strong LL, Von Korff M, Saunders K, Moore JE: **Cost-effectiveness of two self-care interventions to reduce disability associated with back pain.** *SPINE* 2006, **31**.
144. Sundberg R, Tunsater A, Palmqvist M, Ellbjar S, Lowhagen O, Toren K: **A randomized controlled study of a computerized limited education program among young adults with asthma.** *RESPIRATORY MEDICINE* 2005, **99**.
145. Swerissen H, Belfrage J, Weeks A, Jordan L, Walker C, Furler J *et al*.: **A randomised control trial of a self-management program for people with a chronic illness from Vietnamese, Chinese, Italian and Greek backgrounds.** *Patient Educ Couns* 2006, **64**.
146. Taylor RS, Watt A, Dalal HM, Evans PH, Campbell JL, Read KLQ *et al*.: **Home-based cardiac rehabilitation versus hospital-based rehabilitation: a cost effectiveness analysis.** *INTERNATIONAL JOURNAL OF CARDIOLOGY* 2007, **119**.
147. Thomas KS, Miller P, Doherty M, Muir KR, Jones AC, O'Reilly SC: **Cost effectiveness of a two-year home exercise program for the treatment of knee pain.** *Arthritis Rheum* 2005, **53**.
148. Trento M, Passera P, Bajardi M, Tomalino M, Grassi G, Borgo E *et al*.: **Lifestyle intervention by group care prevents deterioration of Type II diabetes: a 4-year randomized controlled clinical trial.** *DIABETOLOGIA* 2002, **45**.
149. Turkington D, Kingdon D, Rathod S, Hammond K, Pelton J, Mehta R: **Outcomes of an effectiveness trial of cognitive-behavioural intervention by mental health nurses in schizophrenia.** *BRITISH JOURNAL OF PSYCHIATRY* 2006, **189**.
150. van der Meer V, van den Hout WB, Bakker MJ, Rabe KF, Sterk PJ, Assendelft WJ *et al*.: **Cost-effectiveness of Internet-based self-management compared with usual care in asthma.** *PLoS One* 2011, **6**.
151. Varma S, McElnay JC, Hughes CM, Passmore AP, Varma M: **Pharmaceutical care of patients with congestive heart failure: interventions and outcomes.** *Pharmacotherapy:The Journal of Human Pharmacology & Drug Therapy* 1999, **19**.
152. Wakabayashi R, Motegi T, Yamada K, Ishii T, Jones RC, Hyland ME *et al*.: **Efficient integrated education for older patients with chronic obstructive pulmonary disease using the Lung Information Needs Questionnaire.** *Geriatrics & gerontology international* 2011, **11**.
153. Wakefield BJ, Ward MM, Holman JE, Ray A, Scherubel M, Burns TL *et al*.: **Evaluation of home telehealth following hospitalization for heart failure: a randomized trial.** *Telemedicine journal & e-health* 2008, **14**.
154. Watson PB, Town GI, Holbrook N, Dwan C, Toop LJ, Drennan CJ: **Evaluation of a self-management plan for chronic obstructive pulmonary disease.** *The European respiratory journal* 1997, **10**.
155. Weinberger M, Tierney WM, Cowper PA, Katz BP, Booher PA: **Cost-effectiveness of increased telephone contact for patients with osteoarthritis. A randomized, controlled trial.** *Arthritis Rheum* 1993, **36**.
156. Whitehurst DGT, Lewis M, Yao GL, Bryan S, Raftery JP, Mullis R *et al*.: **A brief pain management program compared with physical therapy for low back pain: results from an economic analysis alongside a randomized clinical trial.** *ARTHRITIS & RHEUMATISM* 2007, **57**.
157. Whooley MA, Stone B, Soghikian K: **Randomized trial of case-finding for depression in elderly primary care patients.** *JOURNAL OF GENERAL INTERNAL MEDICINE* 2000, **15**.
158. Willmott L, Harris P, Gellaitry G, Cooper V, Horne R: **The effects of expressive writing following first myocardial infarction: A randomized controlled trial.** *Health Psychology* 2011, **30**.
159. Wolf AM, Conaway MR, Crowther JQ, Hazen KY, L Nadler J, Oneida B *et al*.: **Translating lifestyle intervention to practice in obese patients with type 2 diabetes: Improving Control with Activity and Nutrition (ICAN) study.** *DIABETES CARE* 2004, **27**.
160. Wootton R, Gramotnev H, Hailey D: **A randomized controlled trial of telephone-supported care coordination in patients with congestive heart failure.** *Journal of Telemedicine & Telecare* 2009, **15**.
161. Yilmaz A, Akkaya E: **Evaluation of long-term efficacy of an asthma education programme in an out-patient clinic.** *RESPIRATORY MEDICINE* 2002, **96**.
162. Yoon R, McKenzie DK, Bauman A, Miles DA: **Controlled trial evaluation of an asthma education programme for adults.** *Thorax* 1993, **48**.
163. de Oliveira MA, Faresin SM, Bruno VF, de Bittencourt AR, Fernandes AL: **Evaluation of an educational programme for socially deprived asthma patients.** *European Respiratory Journal* 1999, **14:** 908-914.
164. Hamann J, Cohen R, Leucht S, Busch R, Kissling W: **Shared decision making and long-term outcome in schizophrenia treatment.** *Journal of Clinical Psychiatry* 2007, **68:** 992-997.
165. Dougherty CM, Thompson EA, Lewis FM: **Long-Term Outcomes of a Telephone Intervention After an ICD.** *Pacing and Clinical Electrophysiology* 2005, **28:** 1157-1167.
166. Hilary Pinnock, Robert Bawden, Stephen Proctor, Stephanie Wolfe, Jane Scullion, David Price *et al*.: **Accessibility, acceptability, and effectiveness in primary care of routine telephone review of asthma: pragmatic, randomised controlled trial.** *Bmj* 2003, **326**.
167. Pinnock H, Bawden R, Proctor S, Wolfe S, Scullion J, Price D *et al*.: **Accessibility, acceptability, and effectiveness in primary care of routine telephone review of asthma: pragmatic, randomised controlled trial.** *British Medical Journal* 2003, **326:** 477-479.
168. Jenkinson CM, Doherty M, Avery AJ, Read A, Taylor MA, Sach TH *et al*.: **Effects of dietary intervention and quadriceps strengthening exercises on pain and function in overweight people with knee pain: randomised controlled trial.** *British Medical Journal* 2009, **339**.

**Details of individual studies – context**

| **Study ID (1st Author and date)** | **Country** | **N** | **Other LTCs excluded (1=yes)** | **Measures of Effectiveness** | **Health Utilization Outcomes** | **Costs Measures/Types** |
| --- | --- | --- | --- | --- | --- | --- |
| Angermann ^204^ | Germany | 715 | 0 | SF-36 | Hospitalizations, inpatient admissions, physician contacts, medication, outpatient visits |  |
| Barnason ^319^ | US | 280 | 0 | MOS SF-36; | Hospitalisations; emergency department visits; health care provider visits for cardiac problems; |  |
| Barton ^158^ | UK | 389 | 1 | Western Ontario and McMaster Universities osteoarthritis index (WOMAC), EQ5D, Quality Adjusted Life Years (QALYs) | Hospitalizations, GP visits, outpatient visits, inpatient admissions, nurse visits, other health professional visits | Total costs, health care visits costs, medication costs, use of other resources related to knee pain (GP, nurse, other health care professional or hospital, inpatient and outpatient visits) at 1 year follow-up |
| Barton ^158^ | UK | 389 | 1 | WOMAC, EQ5D, SF-36 | Hospitalizations, GP visits, outpatient visits, nurse visits, other health professional visits | Total costs, health care visits costs, medication costs |
| Barton ^158^ | UK | 389 | 1 | WOMAC, EQ5D, SF-36 | Hospitalizations, GP visits, outpatient visits, nurse visits, other health professional visits | Total costs, health care visits costs, medication costs |
| Bauer ^266^ | US | 330 | 0 | SF-36; Mental Health Collaborative Study instrument | Hospitalizations; medication | Total, hospitalization, inpatient, outpatient |
| Bauml ^267^ | Germany | 236 | 0 | Lancashire Quality of Life Profile (Z-Scale); Brief Psychiatric Rating Scale (BPRS); Global Assessment of Functioning (GAF) | Hospital days, hospitalizations; medication |  |
| Beck ^285^ | US | 221 | 0 | SF-36 | CHCC visits, emergency department visits other visits, calls to nurse and doctor, Exams/tests, hospitalizations | Script costs, total costs, hospitalization costs, intervention costs, chcc group visits |
| Beckerman ^168^ | Israel | 42 | 0 | St. George’s Respiratory Questionnaire (SGRQ); modified Borg scale | Hospital days; hospitalizations; primary care consultations |  |
| Behnke ^169^ | Germany | 26 | 0 | Chronic Respiratory Disease Questionnaire (CRQ); modified Borg scale; Baseline Dyspnoea Index/ Transition Dyspnoea Index (BDI/TDI) | Hospitalisations; medication |  |
| Bocchi ^206^ | Brazil | 350 | 1 | The Minnesota Living with Heart Failure Questionnaire (MLHFQ) | Hospitalisations; hospital days; emergency care; medical treatment |  |
| Bosmans ^141^ | Netherlands | 145 | 1 | PRIMary care Evaluation of Mental Disorders (PRIME-MD); EQ5D; Montgomery Asberg Depression Rating Scale (MADRS); QALYs | Hospitalizations, outpatient, inpatient, primary care, medication, supportive care, direct non healthcare | Total, hospitalization, primary care, secondary care, supportive care, direct non health care, psychotropic medication, intervention |
| Bosmans ^140^ | Netherlands | 151 | 0 | Hopkins Symptom Checklist (SCL). | GP visits, specialist visits, out of work, tests, medication | Total costs, primary care costs, secondary care costs, medication, intervention, patient costs |
| Bouvy ^207^ | Netherlands | 152 | 0 | Dartmouth Primary Care Cooperative Information Project/World Organization of National Colleges, Academies, and Academic Associations of General Practice/Family Physicians (COOP-WONCA); Minnesota Heart Failure Questionnaire (MHFQ) | Total number of hospitalisations, planned re-admissions, other hospital admission, medication |  |
| Boxall ^170^ | Australia | 60 | 0 | CRQ (No CRQ used); SGRQ, modified Borg scale, Bartel Activities of Daily Living Index, Short Portable Mental Health Status Questionnaire (SPMSQ) | Hospitalisations, average length of stay at readmission |  |
| Brotons ^208^ | Spain | 283 | 0 | Minnesota Living with heart Failure (MLHFQ) | Hospitalisations; medication |  |
| Brun ^260^ | France | 74 | 0 | French translation of Nottingham Health Profile (NHP); French translation of Diabetes Quality of Life (DQOL) | Hospital admissions; number of outpatient consultations with GP + specialists; medication | Total costs |
| Bulthuis ^148^ | Netherlands | 85 | 1 | SF-36, Health Assessment questionnaire (HAQ), McMaster Toronto Arthritis Patient Preference Disability Questionnaire (MACTAR); QALYs | Hospitalisation, primary care, outpatient, specialist visits, medication, professional domestic care, other paramedical help | Total, hospitalisation, primary care, inpatient, outpatient, specialist, paramedical, alternative, aids used, medication, patient costs, absenteeism, domestic help, formal care, informal care, intervention |
| Capomolla ^136^ | US | 235 | 0 | QALYs | Hospitalisations; medication | Total costs, pharmacological costs, case management costs |
| Castro ^171^ | US | 96 | 0 | Asthma Quality of Life Questionnaire (AQOL) | Hospital readmissions; hospital days; ER visits; healthcare provider visits; medications | Total costs, hospitalizations, emergency department visits, healthcare provider visits, nurse/paid caregiver, tests, asthma medication, loss of productivity/time, intervention costs, nonprofessional/other paid help, unpaid caregiver costs. |
| Clark ^172^ | US | 808 | 0 | Mini Asthma Quality of Life | Hospitalisations; emergency department visits; unscheduled & scheduled visits to clinic, medication |  |
| Clarke ^268^ | US | 255 | 0 | PCS and MCS SF-12; Centre for Epidemiological Studies Depression Scale (CES-D) | Mental Health Outpatients visits, medication, General Health Services Outpatient Visits |  |
| Clarke ^268^ | US | 255 | 0 | SF-36, CES-D | Outpatients visits, medication |  |
| Cline ^209^ | Sweden | 206 | 1 | Quality of life in Heart Failure Questionnaire; Nottingham health profile; patients' global self-assessment | Hospitalisations; hospital days, days to readmission; outpatient visits; medication | Doctor visits, hospitalizations, intervention, total costs |
| Coull ^211^ | UK | 320 | 0 | SF-36; HAD-Depression, HAD-Anxiety | medication, use of secondary care health services. |  |
| Coultas ^173^ | US | 151 | 0 | MOS short form (SF-36); SGRQ; CES-D questionnaire; brief symptom inventory (BSI-18) | Hospitalisations (lung disease); emergency department visits; GP visits; medication; Hospitalisations (other diseases); emergency department visits; GP visits; |  |
| Coultas ^173^ | US | 151 | 0 | MOS short form (SF-36); SGRQ; CES-D questionnaire | Hospitalisations; ED visits; GP visits |  |
| Davidson ^212^ | Australia | 105 | 0 | Heart Failure Needs Assessment Questionnaire (HFNAQ); Minnesota Living with Heart Failure Questionnaire (MLWHFQ); New York Heart Association Classification (NYHA) | Hospitalisations; medication |  |
| Davies ^265^ | UK | 300 | 0 | Audit of Diabetes Dependent Quality of Life (ADDQoL) | GP contacts, other contacts, readmissions, referrals to community Diabetes Specialist Nurse service, time away from normal activities, time in days to readmission, frequency of readmission, hospital length of stay | Hospital length of stay costs |
| de la Porte ^213^ | Netherlands | 240 | 1 | SF-36; MLHFQ; New York Heart Association Classification (NYHA) | Hospitalisations; days in hospital, medication; outpatient visits | Total costs, hospitalizations, days in hospital, outpatient clinic costs, intervention, total patient costs |
| Den Boer ^269^ | UK | 151 | 1 | World Health Organization Quality of Life Assessment (WHOQoL-Bref), Symptom Checklist-90 (SCL-90) (depression, anxiety), Beck Depression Inventory (BDI), State-Trait Anxiety Inventory (STAI) | Contacts with specialists (psychiatrist, other mental health caregivers, including and excluding) cognitive self-help therapist |  |
| De Oliveira ^174^ | Brazil | 52 | 0 | Modified quality of Life questionnaire (Juniper et al, 1992; Jones et al, 1992) | Hospital admissions, emergency department visits, medication |  |
| Dekker ^214^ | US | 41 | 0 | BDI-II; Crandell Cognitions Inventory (CCI); MLHFQ | Hospitalisations; emergency department visits |  |
| DeWalt ^215^ | US | 605 | 0 | Improving Chronic Illness Care Evaluation Heart Failure Symptom Scale (HFQOL); Short-test of Functional Health Literacy in Adults (S-TOFHLA); NYHA | Hospitalisations; emergency department visits |  |
| DeWalt ^216^ | US | 127 | 0 | Modified Minnesota Living with Heart Failure Questionnaire (MLHFQ) | Hospitalisations; reason for admissions (cardiac), medication |  |
| Doughty ^217^ | New Zealand | 197 | 0 | MHFQ | Hospital days, hospitalizations, readmissions for worsening heart failure |  |
| Dougherty ^218^ | US | 168 | 0 | SF-12, CES-D, STAI-Anxiety | Hospital visits, emergency department visits, clinic visits |  |
| Druss ^270^ | US | 80 | 0 | MOS/MCS SF-36 | Primary care visits |  |
| Dunagan ^219^ | US | 151 | 1 | SF-12 not 36, MLHFQ, BDI, NYHA | Hospitalizations; hospital admissions for heart failure; hospital encounters; hospital days; emergency department visits | Hospital costs |
| Dunn ^271^ | US | 101 | 1 | Self-Control Questionnaire for Depression (SCQD), CAPS Davidson Traumatic Stress Scale (DTSS); Hamilton Depression Scale (HAMD); BDI-II; Global Severity Index of Brief Symptom Inventory (BSI GSI); the Addiction Severity Index (ASI) | Hospitalizations; outpatient visits; clinic visits; medication | Discharges, visits, inpatient, outpatient, pharmacy |
| Dunbar ^220^ | US | 246 | 1 | BDI-II, STAI-Anxiety, Duke Activity Status Inventory (DASI) | Hospitalizations, emergency department visits, calls to providers, missed work for any reason |  |
| Dunbar ^220^ | US | 246 | 1 | BDI-II, STAI-Anxiety | Hospitalizations, emergency department visits, calls to providers, missed work for any reason |  |
| Eaton ^175^ | New Zealand | 97 | 1 | BMI, airflow obstruction, dyspnoea and exercise capacity (BODE) Index; Chronic Respiratory Questionnaire Self-Administered (CRQ-SA); SF-36; HADs | Hospitalisations; Time to first COPD-related readmission; Hospital days, emergency department visits (emergency departments or primary care) ; number of inpatient admissions |  |
| Gallefoss ^125^ | Norway | 78 | 0 | HRQoL; SGRQ | Monthly GP visits; Medication; Specialist doctor visits; Hospitalisations | Total costs, hospitalization costs, physician costs, travel costs, patient costs, intervention costs, time cost for those employed/ not employed, medication |
| Gesica ^221^ | UK | 1518 | 0 | Minnesota Heart Failure Questionnaire, NYHA | Hospitalizations, medication |  |
| Gillett ^133^ | UK | 824 | 0 | EQ-5D; QALYs | Medication; healthcare resources (in terms of, GP, nurse, physiotherapist, podiatrist, dietician, optician) | Total costs, primary care visits costs, other healthcare resource visits costs, intervention costs, medication costs, remaining lifetime discounted costs, patient costs |
| Goldberg ^222^ | US | 180 | 0 | MOS SF-36 (error should be SF-12), Medical Outcomes Health Distress Scale, MLHFQ | Hospitalizations, emergency department visits, |  |
| Graves ^163^ | Australia | 432 | 0 | SF-36 |  | Total costs |
| Griffiths ^286^ | UK | 47.6% | 0 | EQ5D, HADs-Anxiety, HAD-Depression | Visits to GP/practice nurse | Total costs, intervention administration costs |
| Groessl ^150^ | US | 363 | 0 | Quality of Well Being Scale (QWB) | Hospitalizations, primary care visits, outpatient, home visit, hospital visits, emergency department visits, total health care contacts | Total cost savings |
| Groessl ^150^ | US | 363 | 0 | Quality of Well Being Scale (QWB) | Hospitalizations, primary care visits, outpatient, home visit, hospital visits, emergency department visits, total health care contacts | Total cost savings |
| Groessl ^150^ | US | 363 | 0 | Quality of Well Being Scale (QWB) | Hospitalizations, primary care visits, outpatient, home visit, hospital visits, emergency department visits, total health care contacts | Total cost savings |
| Gruffydd ^176^ | UK | 174 | 0 | ACQ; Mini-AQLQ | Routine & non-routine contacts; length of inpatients stays, respiratory secondary care contacts; medication | Total costs, inpatient stays, routine consultations, medication, non-routine care |
| Guell ^177^ | Spain | 30 | 0 | Modified dyspnea in daily activities scale; Spanish CRQ; modified Borg Scale | Hospitalisations, medication |  |
| Haas ^253^ | UK | 109 | 1 | MOS SF-36, Modified Von Korff (MVK) scales | Doctor visits, other professionals, medication |  |
| Hamman ^272^ | Germany | 107 | 0 | Global Assessment of Functional Skills; Clinical Global Impressions Scale | Hospitalizations, medication |  |
| Handley ^135^ | US | 226 | 0 | SF-12; QALYs | Hospital days | Total costs; intervention costs; patient costs |
| Hanssen ^223^ | Norway | 288 | 0 | MOS/PCS SF 36 | Hospitalizations, days off work |  |
| Henderson ^165^ | UK | 965 | 0 | EQ-5D; QALYs; ICECAP-O; SF-36, short form Centre for Epidemiologic Depression Scale (CESD-10), Brief STAI; | Hospital use; Community health services/ Primary care use; mental health services use; community care services; care home respite; day services; medication | Total costs, hospital costs, primary care costs, mental health services costs, home care costs, community care costs, day care services costs, medication costs, intervention costs |
| Hermiz ^178^ | Australia | 177 | 0 | SGRQ | Hospitalisations; emergency department visits; primary care visits; GP prescribed drugs; contact with community nurse |  |
| Hernandez ^179^ | Spain | 222 | 1 | SGRQ; SF-12 | Hospitalisations; emergency department visits; inpatient stays - change to inpatient readmissions; hospital days | Emergency department visits; outpatient visits; primary care visits; social support visits; nurse home visits; medication; telephone support call costs; health transport costs; patient costs |
| Holland ^224^ | UK | 293 | 0 | EQ5D; Minnesota Heart Failure Questionnaire (MLHFQ); Health Visual Analogue Scale (VAS) | Hospitalizations, GP visits, GP home visits, nurse visits, nurse home visits, GP phone calls, nurse/other Phone calls, medication |  |
| Hurley ^151^ | UK | 418 | 1 | EQ5D; QALYs; Western Ontario and McMaster Universities Osteoarthritis Index (WOMAC-func) | Hospitalizations, inpatient, outpatient, GP visits, emergency department, specialist, social services, medication, informal care inputs. | Total, primary care, secondary care, medication, informal care expenses, time off work, intervention costs; Knee rehabilitation; outcome costs |
| Hurley ^151^ | UK | 418 | 1 | EQ5D, Western Ontario and McMaster Universities Osteoarthritis Index (WOMAC-func); | Hospitalizations, inpatient, outpatient, GP, emergency department, specialist, social services, medication informal care inputs. | Total costs, intervention costs |
| Hurley ^151^ | UK | 418 | 1 | EQ5D, Western Ontario and McMaster Universities Osteoarthritis Index (WOMAC-func) | Hospitalizations, inpatient, outpatient, GP, emergency department, specialist, social services, medication informal care inputs. | Total costs, intervention costs |
| Jansa ^261^ | Spain | 40 | 1 | SF-12, Spanish Diabetes Quality of Life Test (DQOL) |  | Intervention costs; Patient costs; Healthcare provider costs |
| Jayadevappa ^225^ | US | 23 | 0 | SF-36, QWB-SA, MLHFQ, CES-D, Perceived Stress Scale (PSS) | Hospitalizations; hospital days |  |
| Jerant ^287^ | US | 415 | 0 | EQ5D, SF-36, HAQ, CES-D | Hospitalizations | Total costs |
| Jerant ^287^ | US | 415 | 0 | EQ5D, SF-36, HAQ, CES-D | Hospitalizations | Total costs |
| Jessep ^159^ | UK | 64 | 1 | WOMAC, EQ5D, HADS-Depression, HADS-Anxiety |  | Total costs, Secondary care (outpatient, emergency department other), primary (GP, nurse, other), medication, intervention |
| Johnson ^254^ | UK | 234 | 0 | EQ5D, QALYs, Roland and Morris Disability Questionnaire (RMDQ), GHQ, VAS |  | Total costs |
| Jolly ^226^ | UK | 597 | 0 | EQ5D VAS, HADS-Depression, HADS-Anxiety | GP visits, nurse visits, rehabilitation, medication |  |
| Jolly ^137^ | UK | 525 | 0 | EQ5D, QALYs, SF-36 (should be SF-12), Global Mood Score (GMS), HADS anxiety, HADS depression | Hospitalizations, hospital days, GP consultations, practice nurse consultations, time off work, medication | Total costs; healthcare costs and societal perspective costs; hospital staff costs; home staff costs; home equipment; patient costs; rehabilitation costs |
| Irvine ^134^ | UK | 177 | 0 | EQ-5D, QALYs, | Hospitalizations; medication; all health care professional contacts | Total costs, intervention costs; trainer costs |
| Karjalainen ^255^ | Finland | 170 | 1 | Generic health-related quality of life (15D), Oswestry Disability Index (ODI) | Visits to physicians, visits to physiotherapist, inpatient rehabilitation, hospital days, sick leave days, medication | Total costs, sick leave costs, health care consumption costs |
| Karjalainen ^255^ | Finland | 170 | 1 | EQ5D, Oswestry disability index | Visits to physicians, physiotherapist, inpatient rehabilitation, hospital days, sick leave days | Total, sick leave |
| Kasper ^227^ | US | 200 | 1 | Minnesota Heart Failure Questionnaire MLHFQ, Duke Activity Status Index, NYHA | Hospitalizations, medication | Inpatient stay costs, outpatient pharmacy costs, intervention costs |
| Katon ^273^ | US | 228 | 0 | SCL depression scale, Sheehan Disability Score (SDS), NEO neuroticism scale |  | Total costs, intervention costs (antidepressant medication, speciality mental health visits, primary care mental health visits, intervention visits)  outpatient depression treatment costs (primary care visits without mental health diagnosis, medication, speciality visits, emergency visits, pharmacy, other outpatient costs), total outpatient non depression costs total outpatient costs, inpatient care (medical & mental health) |
| Katon ^274^ | US | 115 | 0 | Anxiety-free days base on Anxiety Sensitivity Index (ASI), Panic Disorder Severity Scale | Intervention visits, other mental health visits, total mental health visits, primary care visits, total primary care & mental health visits, outpatient visits | Total outpatient costs, total mental health costs, total 1-year health service costs, non-mental health primary care, total outpatient non-mental health, total outpatient, inpatient, psychiatric medication, intervention visits, mental health visits |
| Katon ^275^ | US | 1801 | 1 | Depression-Free days, QALYs based on 20-item Hopkins Symptom Checklist (HSCL-20) |  | Grand total health care costs, Total outpatient costs, primary care, outpatient mental health, other outpatient services, inpatient mental health services, inpatient services, antidepressant medications, other medication, intervention costs |
| Katon ^143^ | US | 232 | 0 | ASI, QALYs, CED-D |  | Total, primary care, speciality, emergency department, psychiatric medication, non-psychiatric medications, laboratory tests, medical procedures, intervention, mental health visits, total ambulatory psychiatric visits, in-patient costs, total ambulatory and in-patient costs |
| Katon ^142^ | US | 214 | 0 | Quality of Life SCIRE quality of life (using not validated, 0 to 10, measure) Patient Global Rating of Improvement for depression scale, SCL-20, PHQ-9 | Hospitalizations, medications |  |
| Kauppinen ^126^ | Finland | 167 | 0 | 15D; St George's Respiratory Questionnaire SGRQ | Use of additional healthcare services, including health centre care, specialist care, emergency care, inpatient care, medication | Total costs, direct costs (health centre care, specialists care, emergency care, inpatient care, medication), indirect costs due to sickness days |
| Kennedy ^164^ | UK | 629 | 0 | EQ-5D EuroQol | Inpatient days, outpatient appointments, GP visits, day care appointments, counsellor visits | Total, inpatient days, outpatient appointments, GP visits, day care appointments, counsellor visits, medication cots, intervention costs, patient costs, |
| Khdour ^127^ | Northern Ireland | 173 | 1 | EQ-5D; QALYs, SGRQ; Self-reported adherence (Morisky); COPD Knowledge questionnaire | Hospitalisations; emergency department; Outpatient; GP scheduled & unscheduled visits; hospital admissions; medication use; hospital pharmacist input | Total specific healthcare resources costs, hospital bed days, GP consultations (scheduled & unscheduled), emergency department visits, medication, intervention costs, total intervention costs, Overall total costs |
| Ko ^180^ | China | 60 | 0 | SF-36, SGRQ, Borg score | Hospital readmission, emergency department attendance |  |
| Koff ^128^ | US | 40 | 0 | SGRQ | Hospitalisations; emergency department visits; radiology services; diagnostic & blood tests | Total costs |
| Koehler ^228^ | Germany | 710 | 0 | SF-36, PHQ-9, NYHA | Hospitalizations (any, heart failure), days off work |  |
| Kroenke ^288^ | US | 250 | 1 | PRIME-MD, HSCL-20, SF-36, HSCL-20, Brief Pain Inventory (BPI), Roland Disability Scale, Graded Chronic Pain scale (GCPS), GAD-7 anxiety | Outpatient visits, primary care visits, medical specialty visits, surgical speciality visits, mental health, other visits, ED visits, hospital days, medication |  |
| Kwok ^229^ | China | 105 | 0 | GHQ, London Handicap Scale | Hospitalisations | Total costs, hospitalization & emergency care, outpatient clinic, community nursing, private doctor, community nurse, travel to clinics/hospital, social services, hospital days |
| Lahdensuo ^181^ | Finland | 122 | 0 | St George's Respiratory Questionnaire SGRQ | Hospitalisations; emergency department visits; unscheduled visits to clinic; medication; days off work |  |
| Lee ^182^ | China | 112 | 0 | GHQ; Barthel Index (BI) | Hospitalisations; hospital days; emergency department visits |  |
| Levitt ^276^ | US | 99 | 0 | Heinrichs Abbreviated Quality of Life Scale (QLS-A), BPRS, Modified Colorado Symptom Index (MCSI) | Hospitalizations |  |
| Levy ^183^ | UK | 211 | 0 | SGRQ | Healthcare utilisation; emergency department visits; routine GP visits; emergency GP visits; routine nurse visits; hospital consultations, medication |  |
| Lewin ^230^ | UK | 176 | 1 | GHQ, HADS-Depression, HADS- anxiety | Hospitalizations, GP visits |  |
| Lewin ^138^ | UK | 192 | 0 | SF-12, Seattle Angina Questionnaire (SAQ), HADS-Depression, HADS-Anxiety | Hospital admissions, emergency department admissions, primary care appointments, outpatient appointments, telephone contacts | Hospital admissions, emergency department admissions, primary care appointments, outpatient appointments, telephone contacts |
| Linton ^256^ | Sweden | 243 | 0 | Outcome Evaluation Questionnaire (OEQ), Pain Catastrophizing Scale, HADS-Depression, HADS-anxiety, Activities of Daily Living Scale | Physician visits, physiotherapist visits, doctor visits, medication, sick leave days | Total, health care visits, intervention, sick leave |
| Linton ^256^ | Sweden | 243 | 1 | EQ5D, SF-36, HADS-Depression, HADS-anxiety | physician visits, physiotherapist visits, doctor visits, medication, sick leave days |  |
| Lopez-Cabezas ^231^ | Spain | 134 | 0 | EQ5D; NYHA | Hospitalizations, inpatient re-admissions, medication | Total costs, hospitalization costs, Intervention costs, patient costs |
| Man ^184^ | UK | 42 | 1 | SGRQ; CRQ; SF-36 | Hospitalisations; hospital days; emergency department visits |  |
| Mancuso ^185^ | US | 296 | 1 | AQLQ; Geriatric Depression Scale | Repeated emergency department visits; access to outpatient care |  |
| Markle-Reid ^232^ | Canada | 101 | 0 | SF-36, CES-Depression, Stroke Impact Scale (SIS-16), Kessler-10, cognitive function (SPMSQ), Reintegration to Normal Living Index (RNLI) |  | Total health services costs, direct costs, indirect costs, |
| McBeth ^257^ | UK | 442 | 1 | Chronic Pain Grade questionnaire (CPG), Vanderbilt Pain Management Inventory (VPMI), GHQ, 7-point, self-rated, clinical global impression change score (validated, untitled scale), SF-36 |  | Incremental total costs |
| McBeth ^257^ | UK | 442 | 1 | 7-point, self-rated, clinical global impression change score, SF-36 |  | Incremental total costs |
| McBeth ^257^ | UK | 442 | 1 | 7-point, self-rated, clinical global impression change score, SF-36 |  | Incremental total costs |
| McDonald ^233^ | Ireland | 98 | 0 | Quality of Life Questionnaire (not validated), NYHA | Hospitalizations, medication |  |
| McLean ^187^ | Canada | 225 | 0 | Juniper questionnaire | Emergency department visits; hospital admissions; medication; visits to primary care; days off school/work | Total costs, Hospitalizations, emergency department visits, medical visits, medication, pharmacy fees, days off work |
| McGeoch ^186^ | New Zealand | 159 | 1 | SGRQ; HADS; COPD-SMI | Hospitalisations; emergency department visits; medication; primary care visits |  |
| McGowan ^262^ | Canada | 321 | 0 | Self-rated health (MOS SF-36) | Emergency department visits; hospital admissions; total number of nights spent in hospital; number of primary care visits |  |
| McWilliam ^289^ | Canada | 298 | 0 | MOS SF-36; Quality of Life Index | Hospitalizations, hospital days, emergency department visits, Utilization-homemaking, Utilization professional services |  |
| CPMMPT ^210^ | UK | 1614 | 0 | SF-36, EQ5D |  | Total costs, usual treatment costs (medicines and NHS visits), Intervention costs, NHS costs (GP and hospital visits), all medication; CHD medication; non-CHD medication |
| Mejhert ^234^ | Sweden | 208 | 1 | Nottingham health profile | Hospitalizations, time to first readmission, length of stay, medication |  |
| Meijer ^152^ | Netherlands | 23 | 0 | Dutch version of SF-36; VAS | Return to work | Total costs, direct medical costs (treatment, medication), direct non-medical costs (expedients), indirect non-medical costs (production losses, loss of time, other costs) |
| Moffett ^258^ | UK | 187 | 1 | Roland disability questionnaire, Aberdeen back pain scale, EQ5D, | Exercise classes, GP visits, physiotherapist visits, chiropractor visits, orthopaedic visits, tests/exams, hospital nights, days off work, equipment | Total costs, exercise classes, GP visits, physiotherapist visits, chiropractor visits, orthopaedic visits, tests/exams, hospital nights, days off work, equipment |
| Monninkhof ^129^ | Netherlands | 248 | 0 | Dutch version SGRQ; EQ-5D QALYs | Physiotherapy visits; hospitalisations; scheduled emergency department visits; emergency department visits; inpatient stays; outpatient visits; GP visits; medication; pharmacist use | Total costs, hospitalization costs, intervention costs, healthcare contact for exacerbation, limited activity days |
| Morcillo ^235^ | Spain | 70 | 0 | SF-36; Charlson Index; Spanish version of Pfeiffers’s Short Portable Mental Status Questionnaire; NYHA | Hospitalizations, emergency department visits | Total costs |
| Moudgil ^188^ | UK | 689 | 0 | AQLQ | Emergency department visits; emergency admissions; primary & secondary healthcare visits; medication; deputising services |  |
| Murphy ^236^ | Ireland | 903 | 1 | SF-12 | Hospitalizations, GP visits, Nurse visits |  |
| Murray ^237^ | US | 314 | 0 | CHFQ; NYHA | hospitalizations, emergency department visits, medication | Total, hospitalization, outpatient costs, inpatient costs, intervention costs, medication costs |
| Naylor ^238^ | US | 239 | 0 | Minnesota Heart Failure Questionnaire MLHFQ; Enforced Social Dependency Scale | Hospitalizations, hospital days, Physician Visits, emergency department visits, home visits | Total, Hospitalizations, Physician Visits, emergency department visits, home visits |
| Niemstro ^160^ | Finland | 204 | 1 | Oswestry Low Back Pain Disability Questionnaire (ODI), VAS, 15D, Depression Questionnaire Score (DEPS) | Physician visits, physiotherapist visits, absence from work | Total costs, physician visits, physiotherapist, absence from work, productivity loss |
| Ninot ^189^ | France | 38 | 1 | French version SGRQ; NHP | Number of hospitalisations & length of hospital admissions; medication | Total costs, hospitalization costs, COPD medication costs |
| Nucifora ^239^ | Italy | 200 | 1 | Minnesota Heart Failure Questionnaire MLHF; NYHA | Hospitalizations, length of hospital stay; unplanned outpatient visits, medication |  |
| Nunez ^251^ | Spain | 100 | 1 | SF-36, WOMAC | GP visits, medication | Costs for GP visits |
| Ojeda ^240^ | Spain | 153 | 0 | MLHFQ; NYHA | Hospitalizations, hospital days inpatient re-admissions, medication |  |
| Patel ^153^ | UK | 812 | 0 | SF-36, WOMAC, HAD-Depression, HAD-anxiety EQ-5D Utility, EQ-5D VAS, QALYs | Hospitalizations, outpatients, physiotherapy, emergency department, occupational therapy, community based services, GP, GP home/surgery visits/calls, social worker, practice nurse visits/calls, home help, informal care, medication | Total costs health & social care, Total costs societal, Health & social costs (excluding intervention), patient/ family/friends costs, indirect costs, social security benefits, intervention costs |
| Penn ^277^ | US | 65 | 0 | Social Functioning Scale (SFS), BDI-II, Beck Cognitive Insight Scale (BCIS) | Hospitalizations, hospital days |  |
| Penn ^278^ | US | 46 | 1 | Quality of Life Scale (QLS), Role Functioning Scale (RFS), Multnomah Community Ability Scale (MCAS), Calgary Depression Scale for Schizophrenia (CDSS) | Hospitalizations, hospital days |  |
| Peters ^259^ | New Zealand | 68 | 0 | Sickness Impact Profile (SIP), McGill Pain Questionnaire (MPQ), Pain Behaviour Checklist (PBC), GHQ, BDI, VAS | Medications, physiotherapist treatment |  |
| Peters ^259^ | New Zealand | 68 | 0 | Sickness Impact Profile, Pain Behaviour Checklist, GHQ, BDI, | medications |  |
| Pinnock ^191^ | UK | 278 | 1 | Juniper mini asthma quality of life questionnaire, Short Q asthma morbidity score | Hospital admissions, emergency department consultations, GP consultations, nurse consultations, outpatient consultations, medication |  |
| Pilotto ^190^ | Australia | 170 | 0 | SGRQ | Hospital admissions, emergency department attendances, attended outpatient department, GP consultations, additional visits to GP, consulted other GP practice, days off work |  |
| Price ^192^ | UK | 1553 | 0 | Mini-AQLQ | Number/ type of healthcare contacts including diagnostic investigations; hospitalisations; medications | Expected total annual cost |
| Pyne ^144^ | US | 395 | 1 | **SF-36 (**should be SF-12), QALYs; SCL-20; Depression Health Beliefs Inventory; Quality of Well-being (QWB); |  | Total healthcare costs, Inpatient total, depression-related inpatient, outpatient, total outpatient medication, patient costs (travel/time), incremental costs |
| Ramachandran ^241^ | India | 50 | 1 | Kansas City Cardiomyopathy Questionnaire (KCCQ); NYHA class | Hospitalizations, emergency department visits, medication |  |
| Rea ^193^ | New Zealand | 135 | 1 | SF-36; CRQ | Hospitalisations; hospital days; medication; emergency department visits |  |
| Reynolds ^277^ | Australia | 25 | 0 | QOLI-Brief Version, Colorado Client Assessment Record (CCAR), | Hospitalizations |  |
| Rich ^242^ | US | 282 | 0 | Chronic Heart Failure Questionnaire CHFQ; NYHA class | Hospitalizations, hospital days, re-admissions, medication | Total costs, hospitalizations, intervention costs, other healthcare costs, caregivers time costs |
| Richardson ^290^ | Canada | 303 | 0 | SF-36, LLFDI, CESD | Hospital days, emergency department visits |  |
| Riegel ^243^ | US | 134 | 0 | EQ5D, MLHFQ, PHQ-9-Depression, NYHA class, Specific Activity Scale | Hospitalizations, hospital days, readmissions | Hospitalization costs |
| Ries ^194^ | US | 172 | 0 | QWB; Rand 36-Item Health Survey; CRQ; UCSD Shortness of Breath Questionnaire; Dyspnea Indices; Centres for Epidemiologic Studies-Depression Scale | Hospitalisations; outpatient visits; number of outpatient telephone calls; emergency department visits |  |
| Rivera ^280^ | US | 203 | 0 | Lehman Quality of Life Inventory; Brief Symptom Inventory | Hospitalizations, individual therapy, group therapy, activity with intervention provider, activity with healthcare professional, outpatient clinic visits, contacts with case management, days of day treatment, primary care, patients/time costs |  |
| Rivera ^280^ | US | 203 | 0 | Lehman Quality of Life Inventory | hospitalizations, outpatient, primary care, patients/time costs |  |
| Roberts ^291^ | Canada | 293 | 0 | Psychosocial Adjustment to Illness Scale (PAIS) |  | Total costs, hospital costs, other health services costs, medication, travel cost, loss of wages costs, Total patient costs, Total direct costs, total indirect costs |
| Roberts ^291^ | Canada | 293 | 0 | PAIS |  | Total health services costs, health services costs, medication, travel cost, loss of wages costs, total direct costs, total patient/family costs, total indirect costs, total annual costs |
| Roelfs ^161^ | Netherlands | 360 | 1 | Quebec Back Pain Disability Scale, EQ-5D | GP visits, physiotherapist, manual therapist, medication, medical specialist (outpatient), alternative therapist, thermal pillow, help from friend, absence from work | Total, direct, indirect |
| Ryan ^195^ | UK | 288 | 1 | Asthma control questionnaire (ACQ); Mini-AQLQ | Hospital admissions; medication; unscheduled practice nurse consultations; consultations with GP; out of hours attendances; emergency department attendances; acute exacerbations | Total costs; total cost of intervention; nursing costs; telemonitoring service costs; total costs of healthcare provision; GP consultations; practice nurse respiratory consultations; secondary care costs; emergency services; medication costs |
| Schermer ^130^ | Netherlands | 193 | 0 | AQLQ | Direct health care such as emergency department visits, hospitalisations; medication; primary care asthma consultations; chest physician consultations | Total costs, hospitalization, emergency department visits, physician consultations, medication, productivity loss, intervention costs |
| Schwarz ^244^ | US | 102 | 0 | MLWHF, CES-D | Hospital readmission, emergency department visits | Total costs of care |
| Seto ^245^ | Canada | 100 | 0 | Minnesota Heart Failure Questionnaire MLHFQ; NYHA class | Hospitalizations, hospital nights, emergency department visits, Clinic visits, medication |  |
| Sevick ^154^ | US | 439 | 0 | WOMAC |  | Total costs, in-centre activities, home visits, adverse events, medical referrals, telephone follow-up costs. |
| Sevick ^154^ | US | 439 | 0 | WOMAC |  | Total costs, in-centre activities, home visits, adverse events, medical referrals, telephone follow-up costs. |
| Seymour ^196^ | UK | 60 | 1 | EQ-5D VAS; CRDQ; SGRQ; Borg scale | Hospitalisations; emergency department visits |  |
| Shelledy ^197^ | US | 166 | 1 | SF-36; SRGQ; Borg score | Hospitalizations; clinic visits; emergency department visits; in-patient days; | Hospitalization costs, emergency department costs |
| Simon ^132^ | UK | 453 | 0 | EQ-5D; QALYs | Medication; primary care visits; emergency department visits; outpatients care; day hospital care; inpatient care; auxiliary health care; private health care | Total costs, primary care, hospital costs, emergency department visits costs, auxiliary health care, intervention costs, medication costs |
| Simon ^132^ | UK | 453 | 0 | EQ-5D | Medication; primary care visits; emergency department visits; outpatients care; day hospital care; inpatient care; auxiliary health care; private health care | Total costs, primary care, hospital costs, emergency department visits costs, auxiliary health care |
| Simon ^147^ | US | 407 | 0 | HDRS | Hospitalizations, outpatient visits, speciality mental health visits, other admissions | Total health services costs, hospitalizations, outpatient, medication, intervention costs, time in treatment costs |
| Simon ^281^ | US | 386 | 0 | SCL-20 | Primary care visits, speciality visits, intervention visits | Total health services costs, total outpatient depression costs, outpatient, specialist care, medication, intervention, primary care, hospitalizations |
| Simon ^282^ | US | 785 | 1 | Psychiatric Status Rating (PSR) scale | Hospitalization, primary care, outpatient, medication, psychotherapy | Total, hospitalization, outpatient, medication, intervention |
| Simon ^145^ | US | 600 | 0 | SCL-90 | Speciality mental health care visits, medication, primary care visits | Total depression treatment costs, Total outpatient costs, total health care plan costs, specialist, primary care, medication, intervention, incremental costs |
| Simon ^145^ | US | 600 | 0 | SCL-90 | Speciality mental health care visits, medication, primary care visits | Total, specialist, primary care, medication, intervention |
| Sinclair ^246^ | UK | 324 | 0 | Quality of Life after Myocardial Infarction Questionnaire, Extended Activities of Daily Living (EADL) Scale | Hospitalizations, outpatient visits |  |
| Sisk ^247^ | US | 406 | 0 | SF-12 physical component score only, Minnesota Heart Failure Questionnaire MLHF | Hospitalizations, nurse management components, medication, emergency department visits |  |
| Soler ^198^ | Spain | 26 | 1 | Spanish SGRQ; modified MRC scale (Dyspnea) | Hospitalisations; primary care visits; visits to research clinic; emergency department visits; admissions to intensive care; length of stay in hospital |  |
| Solomon ^252^ | US | 178 | 0 | SF-36 MOS, Modified Health Assessment Questionnaire | Hospitalisations, emergency department visits, GP visits, medication |  |
| Strong ^162^ | US | 255 | 0 | Roland Disability Questionnaire |  | Total, primary care visits, specialist visits, emergency department visits, alternative therapist visits, physical therapy visits, tests/exams, medication, intervention, incremental costs |
| Strong ^162^ | US | 226 | 0 | Roland Disability Questionnaire, SF-36 |  | Total, primary care visits, specialist visits, emergency department visits, alternative therapist visits, tests/exams, medication |
| Sundberg ^199^ | Sweden | 97 | 0 | Swedish Living with Asthma Questionnaire | Hospital admissions; unscheduled visits; medication |  |
| Swerissen ^292^ | Australia | 320 | 1 | Self-rated health, health distress, disability, depression | Hospital department, GP, specialist medical practitioner, allied health professional, mental health professional, emergency department visits |  |
| Taylor ^139^ | UK | 230 | 1 | MACNEW, HADS-Depression, HADS-Anxiety, EQ-5D QALYs | Hospitalizations, hospital nights, primary care consultations, tests, medication, home-based rehabilitation visits | Total costs, hospitalizations, primary care, secondary, medication, tests, hospital equipment, hospital rehabilitation costs, patient costs, hospital staff costs, staff travel costs, home costs |
| Thomas ^155^ | US | 786 | 0 | SF-36, WOMAC, HADS-Depression, HADS-anxiety |  | Total, primary care, secondary care & primary care, intervention costs |
| Trento ^263^ | Italy | 112 | 0 | Modified, Italian, DQOL | Medication; hypoglycaemic treatment; retinopathy | Total costs, transportation costs, opportunity costs, staff costs, pharmaceutical costs, patient costs, total direct costs, other costs |
| Turkington ^283^ | UK | 422 | 0 | Depression Montgomery–  Asberg Depression Rating Scale (MADRS); Comprehensive Psychopathology Rating Scale (CPRS); Psychotic Symptom Rating Scales (PSYRATS) | Hospital days, readmissions, medication |  |
| van der Meer ^131^ | Netherlands | 200 | 1 | EQ-5D; EQ-5D VAS QALYs; Asthma Control Questionnaire | All contact with health care professionals; emergency department visits; hospital admissions; medication | Total health care costs; Productivity costs; total societal costs; hospitalization costs; Intervention costs; medication; other healthcare costs |
| Varma ^248^ | Ireland | 83 | 1 | SF-36, Minnesota Heart Failure Questionnaire | Hospitalizations, emergency department visits |  |
| Wakabayashi ^200^ | Japan | 102 | 1 | SGRQ; Mini-Mental State Examination (MMSE); Instrumental Activities of Daily Living Questionnaire (ADL); LINQ score; Modified MRC Dyspnea Scale (MMRC); Bode Index | Hospitalisations; emergency department visits |  |
| Wakefield ^249^ | US | 148 | 0 | Minnesota Living with Heart Failure Questionnaire; NYHA class; Mini-Mental Status Examination; Geriatric Depression Scale (GDS) | Hospitalizations; hospital days; urgent care visits; intervention contacts |  |
| Wakefield ^249^ | US | 148 | 0 | Minnesota Living with Heart Failure Questionnaire | Hospitalizations |  |
| Watson ^201^ | New Zealand | 56 | 1 | SGRQ | Hospitalisations; medication; hospital specialist visits; pharmacist visits; primary care visits (GP/PN) |  |
| Weinberger ^156^ | US | 191 | 1 | Arthritis Impact Measurement Scales (AIMS); self-rated health status (validated measure) | Intervention contacts | Inpatient, outpatient costs, emergency department costs, total costs |
| Whitehurst ^157^ | UK | 402 | 1 | EQ5D,QALYs | Treatment sessions, primary care contacts, inpatient episodes, outpatient attendances, other healthcare professionals, medication. | Total costs, treatment sessions, primary care contacts, inpatient episodes, outpatient attendances, other healthcare professionals, medication. |
| Whooley ^284^ | US | 331 | 0 | 15-item Geriatric Depression Scale GDS | Hospitalizations, clinic visits |  |
| Willmott ^250^ | UK | 179 | 1 | SF-36; return to work | Combined GP & hospital visits, medication; attendance at cardiac rehabilitation |  |
| Wolf ^264^ | US | 147 | 0 | SF-36 (MOS) | Medication; intervention sessions |  |
| Wootton ^293^ | Australia | 525 | 0 | SF-36, EQ5D | Hospital treatment, pharmacy, other treatment services, medical treatment community nursing treatment, allied health treatment | Total, hospital treatment, pharmacy, other treatment services, medical treatment community nursing treatment, allied health treatment |
| Yilmaz ^202^ | Turkey | 80 | 0 | Asthma quality of life questionnaire (AQOL) | Emergency department visits; hospital admissions; medication |  |
| Yoon ^203^ | Australia | 76 | 1 | Psychosocial disturbance questionnaire | Hospitalisations; emergency department visits; missed work/school |  |

**Details of individual studies - patients**

| **Study** | **Long term conditions** | **Males** | **Mean Age (Mean)** | **Eligible patients who did not take part** |
| --- | --- | --- | --- | --- |
| Angermann ^204^ | Heart failure | 31% | 69.4 | 21% |
| Barnason ^319^ | Chronic heart failure | 83% | 71 | 17% |
| Barton ^158^ | Knee pain | 35% | 61.5 | 32% |
| Barton^158^ | Knee pain | 35% | 61.5 | 32% |
| Barton ^158^ | Knee pain | 35% | 61.5 | 32% |
| Bauer ^266^ | Bipolar disorder | 91% | 46.6 | 33% |
| Bauml ^267^ | Psychosis | 43% | 34 | 15% |
| Beck ^285^ | Heart disease, lung disease, joint disease, diabetes | 36% | 75 | 50% |
| Beckerman ^168^ | Chronic obstructive pulmonary disease | 71.4 | 66.9 |  |
| Behnke ^169^ | Chronic obstructive pulmonary disease | 75% | 69 |  |
| Bocchi ^206^ | Chronic heart failure | 64% | 52 |  |
| Bosmans ^141^ | Depression | 46% | 64.7 | 46% |
| Bosmans ^140^ | Depression | 31% | 43 | 29% |
| Bouvy ^207^ | Heart failure | 60% | 70.2 |  |
| Boxall ^170^ | Chronic obstructive pulmonary disease | 65% | 76 |  |
| Brotons ^208^ | Chronic heart failure | 44% | 76 | 37% |
| Brun ^260^ | Type 2 diabetes | 100% | 60.6 |  |
| Bulthuis ^148^ | Arthritis | 20% | 69 | 25% |
| Capomolla ^136^ | Chronic heart failure | 84% | 56 |  |
| Castro ^171^ | Asthma | 15% | 38 |  |
| Clark ^172^ | Asthma | 0% | 49 | 32% |
| Clarke ^268^ | Depression | 24% | 45 | 12% |
| Clarke ^268^ | Depression | 24% | 45 | 12% |
| Cline ^209^ | Chronic heart failure | 52% | 76 |  |
| Coull ^211^ | Ischaemic heart disease | 60% | 67.4 | 19% |
| Coultas ^173^ | Chronic obstructive pulmonary disease | 54% | 69 | 23% |
| Coultas ^173^ | Chronic obstructive pulmonary disease | 54% | 69 | 23% |
| Davidson ^212^ | Chronic heart failure | 60% | 74 | 33% |
| Davies ^265^ | Type 1 or 2 diabetes | 55.3% | 63.4 median | 41% |
| de la Porte ^213^ | Chronic heart failure | 79% | 71 | 49% |
| Den Boer ^269^ | Depression or anxiety disorder | 47% | 41.9 | 17% |
| De Oliveira ^174^ | Asthma | 15% | 38 |  |
| Dekker ^214^ | Chronic heart failure | 43% | 64 | 37% |
| DeWalt ^215^ | Chronic heart failure | 52% | 60 | 30% |
| DeWalt ^216^ | Chronic heart failure | 41% | 62 | 3% |
| Dougherty ^218^ | Chronic heart failure | 73.8% | 65 |  |
| Doughty ^217^ | Heart failure | 60% | 73.5 |  |
| Druss ^270^ | Mental Illness | 26% | 48.4 | 29% |
| Dunagan ^219^ | Heart failure | 47% | 69.4 | 45% |
| Dunn ^271^ | Post-Traumatic Stress disorder and Depression | 100% | 55 | 40% |
| Dunbar ^220^ | Patients with implantable cardioverter defibrillator | 70.1% | 58.4 | 48% |
| Dunbar ^220^ | Patients with implantable cardioverter defibrillator | 70.1% | 58.4 | 48% |
| Eaton ^175^ | Chronic obstructive pulmonary disease | 42% | 70 | 58% |
| Gallefoss ^125^ | Asthma | 21% | 44 |  |
| Gesica ^221^ | Chronic heart failure | 68.9% | 65.2 | 72% |
| Gillett ^133^ | Type 2 diabetes | 26 | 61 |  |
| Goldberg ^222^ | Heart failure | 65.5% | 60.2 |  |
| Graves 2009 ^163^ | Diabetes, hypertension | 40.3% | 57.8 | 36.6% |
| Griffiths ^286^ | Diabetes, cardiovascular disease, respiratory, arthritis | 42% | 48 | 76% |
| Groessl ^150^ | Arthritis | 35.8% | 69 | 75% |
| Groessl ^150^ | Arthritis | 35.8% | 69 | 75% |
| Groessl ^150^ | Arthritis | 35.8% | 69 |  |
| Gruffydd ^176^ | Asthma | 40% | 50 |  |
| Guell ^177^ | Chronic obstructive pulmonary disease | 100% | 66 | 8% |
| Haas ^253^ | Low back pain | 22.2% | 75.5 |  |
| Hamman ^272^ | Psychosis | 52% | 38 |  |
| Handley ^135^ | Type 2 diabetes | 55.8 | 45.2 | 14% |
| Hanssen ^223^ | Acute Myocardial Infraction | 76.5% | 60.9 | 28% |
| Henderson ^165^ | Heart failure, chronic obstructive pulmonary disease, diabetes | 60% | 70.6 |  |
| Hermiz ^178^ | Chronic obstructive pulmonary disease | 46% | 67 |  |
| Hernandez ^179^ | Chronic obstructive pulmonary disease | 97% | 71 | 4% |
| Holland ^224^ | Heart failure | 63.2% | 76.4 | 23% |
| Hurley ^151^ | Chronic knee pain | 29.7% | 66 | 62% |
| Hurley ^151^ | Chronic knee pain | 29.7% | 66 | 62% |
| Hurley ^151^ | Chronic knee pain | 29.7% | 66 | 62% |
| Jansa ^261^ | Type 1 diabetes | 68.8% | 23 | 20% |
| Jayadevappa ^225^ | Heart failure | 20% | 63.8 | 88% |
| Jerant ^287^ | Arthritis, asthma, chronic obstructive pulmonary disease, congestive heart failure, depression, and/or diabetes mellitus | 25% | 60.1 | 32% |
| Jerant ^287^ | Arthritis, asthma, chronic obstructive pulmonary disease, congestive heart failure, depression, and/or diabetes mellitus | 25% | 60.1 | 2% |
| Jessep ^159^ | Knee pain | 37.1% | 67 | 2% |
| Johnson ^254^ | Low back pain | 42% | 48.5 | 39% |
| Jolly ^226^ | Myocardial infraction or angina | 74% | 64 |  |
| Jolly ^137^ | Myocardial infarction or coronary revascularisation | 76% | 61.8 | 57% |
| Irvine ^134^ | Type 2 diabetes | 50.8 | 58.7 | 15% |
| Karjalainen ^255^ | Low back pain | 40% | 43 | 4% |
| Karjalainen ^255^ | Low back pain | 40% | 43 | 4% |
| Kasper ^227^ | Chronic heart failure | 56.1% | 63.7 | 12% |
| Katon ^273^ | Depression | 17% | 46.7 | 32% |
| Katon ^274^ | Panic disorder | 36% | 41.9 | 76% |
| Katon ^275^ | Depressive disorders | 34% | 71.4 | 16% (screened), 12% (referred) |
| Katon ^143^ | Panic disorder | 34% | 41.9 | 76% |
| Katon ^142^ | Depression + diabetes or coronary heart disease | 44% | 56.3 | 9% |
| Kauppinen ^126^ | Asthma | 42.70% | 44 |  |
| Kennedy ^164^ | Mixed | 30.4% | 55.3 | 23% |
| Khdour ^127^ | Chronic obstructive pulmonary disease | 45% | 67 |  |
| Ko ^180^ | Chronic obstructive pulmonary disease | 96.7% | 73.8 | 26% |
| Koff ^128^ | Chronic obstructive pulmonary disease | 50% | 65 |  |
| Koehler ^228^ | Chronic heart failure | 82% | 66.9 |  |
| Kroenke ^288^ | Depression and Pain | 50% | 55.8 | 25% |
| Kwok ^229^ | Heart failure | 45% | 76.8 |  |
| Lahdensuo ^181^ | Asthma | 47.5 | 43 |  |
| Lee ^182^ | Chronic obstructive pulmonary disease | 49% | 80 |  |
| Levitt ^276^ | Serious mental health | 64% | 55 |  |
| Levy ^183^ | Asthma | 43% | 40 | 33% |
| Lewin ^230^ | Acute myocardial infarction | 72.7% | 56.3 | 11% |
| Lewin ^138^ | First implantable cardioverter defibrillator implantation | 74% | 58.7 | 12% |
| Linton ^256^ | Spinal pain | 29% | 45 | 37% |
| Linton ^256^ | Spinal pain | 26% | 44 | 37% |
| Lopez-Cabezas ^231^ | Heart failure | 46.9% | 76.1 |  |
| Man ^184^ | Chronic obstructive pulmonary disease | 38% | 71 | 15% |
| Mancuso ^185^ | Asthma | 23% | 43 | 36% |
| Markle-Reid ^232^ | Stroke | 62% | 70.6 | 66% |
| McBeth ^257^ | Chronic widespread pain | 30.3% | 56.3 | 50% |
| McBeth ^257^ | Chronic widespread pain | 30.3% | 56.3 | 50% |
| McBeth ^257^ | Chronic widespread pain | 30.3% | 56.3 | 50% |
| McDonald ^233^ | Heart failure | 70.2% | 70.8 | 54% |
| McLean ^187^ | Asthma | 37% | 48 | 10% |
| McGeoch ^186^ | Chronic obstructive pulmonary disease | 67% | 72 | 7% |
| McGowan ^262^ | Type 2 diabetes | 45 | 59 |  |
| McWilliam ^289^ | Mixed | 36% |  |  |
| CPMMPT ^210^ | Coronary heart disease | 70.6% | 68.8 | 58% |
| Mejhert ^234^ | Heart failure | 59% | 75.7 | 27% |
| Meijer ^152^ | Non-specific upper extremity musculoskeletal disorders | 60.9% | 37.9 | 11% |
| Moffett ^258^ | Low back pain | 44% | 42.6 |  |
| Monninkhof ^129^ | Chronic obstructive pulmonary disease | 84% | 65 |  |
| Morcillo ^235^ | Heart failure | 56% | 76.3 |  |
| Moudgil ^188^ | Asthma | 47% | 35 | 43% |
| Murphy ^236^ | Coronary heart disease | 70% | 66.5 | 30% |
| Murray ^237^ | Heart failure | 33.9% | 62.6 | 3% |
| Naylor ^238^ | Heart failure | 44% | 75.6 | 63% |
| Niemstro ^160^ | Low back pain | 47% | 36.7 | 3% |
| Ninot ^189^ | Chronic obstructive pulmonary disease | 78% | 61 | 16% |
| Nucifora ^239^ | Heart failure | 62% | 73 |  |
| Nunez ^251^ | Osteoarthritis | 35% | 69.5 | 4% |
| Ojeda ^240^ | Heart failure | 62% | 65 | 22% |
| Patel ^153^ | Arthritis | 31% | 68.7 | 63% |
| Penn ^277^ | Schizophrenia | 49% | 39.6 | 21% |
| Penn ^278^ | First-episode psychosis | 61% | 20.9 | 39% |
| Peters ^259^ | Chronic pain | 43.7% | 43.9 | 38% |
| Peters ^259^ | Chronic pain | 43.7% | 43.9 | 38% |
| Pinnock ^191^ | Asthma | 41% | 56.4 | 53% |
| Pilotto ^190^ | Asthma | 47.8% | 49.7 | 53% |
| Price ^192^ | Asthma | 41% | 48 | 10% |
| Pyne ^144^ | Depression | 89% | 60 | 40% |
| Ramachandran ^241^ | Heart failure | 76% | 45.8 | 6% |
| Rea ^193^ | Chronic obstructive pulmonary disease | 41% | 68 | 23% |
| Reynolds ^277^ | Mental Illness (bipolar, schizophrenia, depression) |  |  | 5% |
| Rich ^242^ | Congestive heart failure | 41% | 78.4 | 18% |
| Richardson ^290^ | Mixed | 62.3% |  | 49% |
| Riegel ^243^ | Heart failure | 50.8% | 72.7 | 40% |
| Ries ^194^ | Chronic obstructive pulmonary disease | 54% | 67 |  |
| Rivera ^280^ | Mental illness | 53% | 36.7 | 37% |
| Rivera ^280^ | Mental illness | 53% | 36.7 | 37% |
| Roberts ^291^ | Mixed | 31% | 43.7 | 40% |
| Roberts ^291^ | Mixed | 31% | 43.7 | 40% |
| Roelfs ^161^ | Low back pain | 3% | 41.5 | 27% |
| Ryan ^195^ | Asthma | 41% | 52 | 27% |
| Schermer ^130^ | Asthma | 42% | 39 | 55% |
| Schwarz ^244^ | Heart failure | 61% | 79.1 | 11% |
| Seto ^245^ | Heart failure | 76% | 52.3 | 46% |
| Sevick ^154^ | Arthritis | 31% | 69 | n/a |
| Sevick ^154^ | Arthritis | 31% | 69 | n/a |
| Seymour ^196^ | Chronic obstructive pulmonary disease | 47% | 65 |  |
| Shelledy ^197^ | Asthma | 22% | 44 | 21% |
| Simon ^132^ | Type 2 diabetes | 55.9 | 66.3 | 44% |
| Simon ^132^ | Type 2 diabetes | 55.9 | 66.3 | 44% |
| Simon ^147^ | Depression | 22% | 45.4 | 31% |
| Simon ^281^ | Depression | 28% | 45.6 |  |
| Simon ^282^ | Bipolar disorder | 31% | 44.3 | 2% |
| Simon ^145^ | Depression | 22% | 44 | 5% |
| Simon ^145^ | Depression | 22% | 44 | 5% |
| Sinclair ^246^ | Myocardial infarction | 53% | 73.8 | 28% |
| Sisk ^247^ | Heart failure | 52.2% | 59.3 | 74% |
| Soler ^198^ | Chronic obstructive pulmonary disease |  | 73 |  |
| Solomon ^252^ | Osteoarthritis, rheumatoid arthritis, or fibromyalgia | 26% | 61 | 12% |
| Strong ^162^ | Back pain |  |  |  |
| Strong ^162^ | Back pain | 50.4% | 49.1 | 12% |
| Sundberg ^199^ | Asthma | 55% | 19 |  |
| Swerissen ^292^ | Mixed | 21% | 65.4 | 35% |
| Taylor ^139^ | Acute myocardial infarction | 80% | 64.3 | 18% |
| Thomas ^155^ | Knee pain | 44.9% | 61.9 | 7% |
| Trento ^263^ | Type 2 diabetes | 34 | 61 |  |
| Turkington ^283^ | Schizophrenia |  |  | 37% |
| van der Meer ^131^ | Asthma | 29% | 37 | 21% |
| Varma ^248^ | Heart failure | 36.6% | 76.4 |  |
| Wakabayashi ^200^ | Chronic obstructive pulmonary disease | 84% | 70 |  |
| Wakefield ^249^ | Heart failure | 98% | 67.2 | 38% |
| Wakefield ^249^ | Heart failure | 98% | 67.2 | 38% |
| Watson ^201^ | Chronic obstructive pulmonary disease | 67% | 67 |  |
| Weinberger ^156^ | Osteoarthritis | 11.4% | 61.1 | 25% |
| Whitehurst ^157^ | Low back pain | 45% | 40.9 | 11% |
| Whooley ^284^ | Depression | 38% | 75.9 | 16% |
| Willmott ^250^ | Myocardial infarction | 83% | 63 | 20% |
| Wolf ^264^ | Type 2 diabetes | 42 | 53.4 |  |
| Wootton ^293^ | Mixed | 54% | 78.1 |  |
| Yilmaz ^202^ | Asthma | 30% | 29 |  |
| Yoon ^203^ | Asthma | 28% |  | 59% |
|  |  |  |  |  |

**Details of individual studies – interventions**

| **Study ID (1st Author and date)** | **Content of Intervention** | **Content of Control** | **Intensity of intervention** | **Follow-up (months)** |
| --- | --- | --- | --- | --- |
| Angermann ^204^ | Nurse-led post discharge disease management intervention addressing individual problems raised by patients, pursuing networking of health care providers and training for caregivers | Usual care | Initial meeting prior to discharge, telephone contacts (weekly for 1st month and at least 1 per month for 5 months)= 2.5 hours the lowest | 6 |
| Barnason ^319^ | Self-management telehealth device + program based on behavioural theory | Usual outpatient care | Daily use x 6 weeks | 6 |
| Barton ^158^ | Dietary intervention plus group-based quadriceps strengthening exercises + individualised reinforcement visits | Leaflet provision | Visits monthly for 6 months and then every other month for 18 months= 15 visits= 7.5 hrs | 6,12,24 |
| Barton ^158^ | Dietary intervention only | Leaflet provision | Visits monthly for 6 months and then every other month for 18 months= 15 visits= 7.5 hrs | 6,12,24 |
| Barton ^158^ | Quadriceps strengthening exercises only | Leaflet provision | 6 phone calls (visits were the same with control)= 30 mins | 6,12,24 |
| Bauer ^266^ | Nurse-led collaborative intervention enhancing patient self-management skills with group psychoeducation; providing clinician decision support with simplified practice guidelines; and improving access to care, continuity of care + information | Usual care | intense-but unclear | 36 |
| Bauml ^267^ | Patient + relatives separate psychoeducational group therapy | Usual care | 4 1-hr weekly sessions + 4 1-hr monthly sessions+ 8 1,5-hr bi-weekly sessions with relatives= 16 sessions | 84 |
| Beck ^285^ | Group outpatient visits | Usual care | Monthly 2hrs and 15 mins outpatient meetings (not sure about the number= 12 sessions (25 hrs0 | 12 |
| Beckerman ^168^ | Long-term inspiratory muscle training in a rehabilitation program | Low load training | Two sessions of 15 min each, six times a week for 12 months. | 12 |
| Behnke ^169^ | Combined hospital, supervised, exercise training group and home-based exercise training at individual intensity | Usual hospital care | 1x treadmill plus 105 mins (5x) walking training at hospital, plus 45 mins (3x) walking training plus 15 mins diary entry per day. | 18 |
| Bocchi ^206^ | Hospital outpatient disease management program including education, monitoring, plus telephone monitoring | Usual outpatient care | 7 sessions | 30 |
| Bosmans ^141^ | General practitioner training on how to implement the disease management program consisting of late-life depression screening (Dutch guidelines), patient education, drug therapy with paroxetine, and supportive contacts. | Usual care | 8 GP sessions= 4 hours the lowest | 12 |
| Bosmans ^140^ | Pharmacist-coaching intervention consisted of three contacts with the pharmacist; a take-home video reviewing important facts on depression and antidepressant treatment. | Usual care | 1 pharmacist session (20 mins) at baseline+ 1 session (14mins) two weeks later + 1 session (13 mins) at three months= 3 session (47 mins) | 6 |
| Bouvy ^207^ | Pharmacist-led intervention on medication compliance in hospitalized/ outpatients with heart failure | Usual care | 1 interview session + 6 monthly contacts | 6 |
| Boxall ^170^ | Home-based individualized program including graduated walking & arm exercises, individual multidisciplinary education sessions & weekly physiotherapist clinic visits | Delayed self-management | Home-based, daily walking/ arm exercises (progressive 1 min - 30 mins), plus diary recording (15 mins), & 270 mins of weekly visits to physiotherapist (9x 30 mins) | 3 |
| Brotons ^208^ | Home-based intensive educational program, including coordination with physician & cardiologist, post hospitalization | Usual care | x12 monthly visits to home plus telephone contacts (15 mins) every 15 days | 12 |
| Brun ^260^ | Structured exercise programme, including education + training at home | Usual care | 8 x 2 hour sessions = 16 hours | 12 |
| Bulthuis ^148^ | 3-week intensive exercise program, individualized + group-based, post hospitalization, for patients with rheumatic diseases at the European Care Residence and Resort “Groot Stokkert,” which offers hotel facilities and professional care for disabled persons | Usual care | 2 75-mins daily physician sessions for 3 weeks +group education programme 2 per week= 36 sessions | 12 |
| Capomolla ^136^ | Day hospital care program including coordination from multidisciplinary staff + care plan for chronic heart failure patients | Usual care |  | 12 |
| Castro ^171^ | Multifaceted intervention, including education, psychosocial support, self-management plan & coordination of care for ‘high-risk’ inpatients with asthma | Usual care |  | 12 |
| Clark ^172^ | Individualized, nurse delivered, telephone counselling, multi-component intervention based on self-regulation theory for women with asthma | Usual care | 225 mins | 12 |
| Clarke ^268^ | Pure self-help Internet site, (Overcoming Depression on the InterNet) offering training in cognitive restructuring using postcard reminders or telephone reminders | Usual care | pure self-management-only 3 reminder postcards were sent | 4,12 |
| Clarke ^268^ | Pure self-help Internet site, (Overcoming Depression on the InterNet) using telephone reminders | Usual care | Pure self-management-only 3 reminder phone calls were made | 4,12 |
| Cline ^209^ | Patients and families educational program on heart failure during hospitalization + discharge & follow up nurse-led outpatient clinic | Usual care | 2 hours, 30 mins | 12 |
| Coull ^211^ | Patient participation in a volunteer mentor-led group with input from cardiac rehabilitation specialists, programme relating to cardiovascular disease, management and self-help based on a person-centred approach | Usual care | 2 hours monthly for a year= 12 2hour sessions | 12 |
| Coultas ^173^ | Nurse-assisted collaborative care or medical management rehabilitation training programme concerning case scenarios of | Usual care | 8 Hours of standardised medical management GOLD training plus initial contact at home & once a month telephone call to patient (30 mins) | 6 |
| Coultas ^173^ | Nurse-assisted collaborative management training | Usual care | 16 Hours of standardised medical management GOLD training, plus collaborative care training, plus initial contact at home & once a month telephone call to patient (30 mins) | 6 |
| Davidson ^212^ | Multidisciplinary, monitored, cardiac rehabilitation exercise program, outpatient clinic & home-based, without pharmacological therapy | Usual care | 30 mins plus 10 mins exercise x 12, plus 45 mins telephone support | 12 |
| Davies ^265^ | Hospital diabetes specialist nursing service consisting of individual structured patient education appropriate to need, and practical management advice including verbal and written case-note feedback to ward-based medical and nursing staff | Usual care | appropriate to need-not clear | 12 |
| de la Porte ^213^ | Intensive combined nurse/ physician clinic following hospital discharge, consisting of education components plus counselling, diet advice (via dietician) & physical examination for patients with heart failure | Usual care | 4 hours, 30 mins | 12 |
| Den Boer ^269^ | Cognitive self-therapy group sessions led by therapists in outpatient clinics for patients with depression and anxiety which aims for patients to become ‘paraprofessionals’ and to conduct sessions with peers | Usual care | 1-3 45mins preparatory sessions+ 3 orientation sessions+5 weekly day-long sessions+weekly self-therapy sessions. | 18 |
| De Oliveira ^174^ | Outpatient asthma education programme, including a treatment plan, for patients with moderate – severe asthma | Usual care | 6 monthly visits +2 1-hr information about asthma sessions | 6 |
| Dekker ^214^ | Brief individualized cognitive therapy program including single session in the hospital plus single telephone support call post discharge for patients with heart failure & depressive symptoms | Usual care | 35 minutes | 3 |
| DeWalt ^215^ | Multisession, literacy sensitive, behavioural self-management program (ongoing telephone based support) for patients with heart failure | Single session group, usual outpatient care | 1 hour, 10 minutes of calls, plus follow-up calls every 2 weeks until necessary | 12 |
| DeWalt ^216^ | Literacy sensitive, self-management program including educational session, picture based self-care materials, and telephone support calls for patients with heart failure | Education pamphlet plus usual care | 1 hour plus 15 mins x8 calls = 3 hours | 12 |
| Dougherty ^218^ | combined education and telephone intervention delivered by trained cardiovascular nurses compared to the usual care | Usual care | 8 sessions x 20 mins= 160 mins=2 hours and 40 mins | 6, 12 |
| Doughty ^217^ | Integrated heart failure management programme, including individualized pharmacological treatment, which took place in hospital-based clinic post discharge & co-ordination of follow-up care between GP and clinic and patient and family | Usual care | 1 initial clinic visit with nurse + 6 weekly visits + 3 (1.5 hrs) group education sessions= 10 visits= 11 hours | 6 |
| Druss ^270^ | Self-care disease management, a manualised, six-session intervention, delivered by mental health peer leaders | Usual care | peer specialist-led 3 sessions | 6 |
| Dunagan ^219^ | Nurse-led telephone disease management involving scheduled telephone calls post discharge by specially trained nurses promoting self-management and guideline-based therapy as prescribed by primary physicians for patients with heart failure. | Usual care | 3 initial telephone nurse contacts + further telephone support based on participants needs | 6, 12 |
| Dunn ^271^ | Self-management therapy for veterans with chronic posttraumatic stress disorder and depression, didactic presentations on depression components, group discussion, in-session exercises for understanding concepts, and weekly homework assignments. | Psycho-education | 14 1.5hrs weekly sessions (same in control)=20hrs | 3-6, 12 |
| Dunbar ^220^ | Nurse-led telephone counselling intervention that included education, symptom management, and coping skills training for patients after insertion of an implantable cardioverter defibrillator to reduce symptoms of depression & anxiety | Usual care | 30 mins initial session + 4 1hour telephone sessions + booster session=5 hours and 30 mins | 6 &12 |
| Dunbar ^220^ | Group counselling intervention that included education, symptom management, and coping skill training | Usual care | 30 mins initial session + 4 1hour telephone sessions + booster session=5 hours and 30 mins | 6 &12 |
| Eaton ^175^ | Inpatient supervised structured exercise program & outpatient rehabilitation program | Usual care, ATS/ERS COPD guidelines | Daily 30 mins of exercise plus 16 hours of supervised exercise training (1 hour sessions of exercise training twice weekly x8 weeks) | 3 |
| Gallefoss ^125^ | Group-based & individual education & counselling programme, including the provision of a written self-management plan in patients with asthma | Usual care | 180 mins | 12 |
| Gesica ^221^ | Nurse-led telephone intervention to educate and monitor worsening heart failure in outpatients | Usual care | 4 phone calls every 14 days + phone calls every 30 days (14 days or 7 days depending on severity= 1 hour and 20 mins | 16 |
| Gillett ^133^ | Structured group education programme for ongoing and newly diagnosed type 2 diabetes | Usual care | 6 Hours (development paper; Davies, 2008) | 12 |
| Goldberg ^222^ | Technology-based heart failure monitoring system for patients with advanced heart failure | Usual care | only instructions were given during the nurse visit | 6 |
| Graves ^163^ | Telephone counselling intervention to improve physical activity and diet. | Usual care | 7 2–2.5-hours sessions scheduled on consecutive weeks lead by 2 volunteers (at least one of them was lay-leader)= 7 sessions (14 hrs) | 3 |
| Griffiths ^286^ | Lay-led, culturally adapted, self-management programme (CDSMP Expert Patient Programme) in a South Asian chronic disease group | Usual care | 6-weekly, 3-hour sessions, and took place in general practices or community centres. The programmes were led by pairs of trained and accredited Bangladeshi lay tutors, who themselves had chronic diseases (mainly diabetes), who acted as facilitators. | 4 |
| Groessl ^150^ | Social support intervention led by staff members, involved unstructured group discussions prompted by weekly task assignments aimed at promoting empathy and sharing of coping techniques between group members with chronic illness. | Non-volunteers to study with diagnosis confirmed | 10 weekly 2-hour meetings followed by 10 monthly 2-hour meetings =20 sessions= 40 hrs | 12,24,36 |
| Groessl ^150^ | The education intervention involved 2-hour presentations by health educators who were paid to participate in the project. | Non-volunteers to study with diagnosis confirmed | 10 weekly 2-hour meetings followed by 10 monthly 2-hour meetings =20 sessions= 40 hrs | 12,24,36 |
| Groessl ^150^ | The combination intervention included both educational classes and social support, with the first hour dedicated to education and the second to social support. During the second hour no staff members were present. | Non-volunteers to study with diagnosis confirmed | 10 weekly 2-hour meetings followed by 10 monthly 2-hour meetings =20 sessions= 40 hrs | 12,24,36 |
| Gruffydd ^176^ | Targeted routine asthma care by nurse-led, telephone delivered, using the Royal College of Physicians three questions, to formulate individualised written asthma action plan | Usual care | 36 mins | 12 |
| Guell ^177^ | Long-term outpatient, pulmonary multicomponent rehabilitation program for patients, including drug regime, breathing re-training, chest physiotherapy, supervised exercise | Usual care | 1 hour session x12 weeks (12 hours), plus 2 hours, 30 mins session x 12 weeks (27.6 hours), plus 30 mins session x 24 weeks (12 hours) = 51.6 hours | 12 |
| Haas ^253^ | Community-based, lay-led, Chronic Disease Self-Management Program for patients with chronic low back pain in older Americans | Wait list control | community-based, 6-week workshop taught by trained lay people. Each weekly class was 2,5 hours= 6 sessions= 15 hrs | 6 |
| Hamman ^272^ | Shared decision making program on antipsychotic drug use consisting of decision aid and a ‘planning talk’ between patient with schizophrenia and hospital physician | Usual care | 1 session for booklet/psychoeducation + 1 physician visit | 6,18 |
| Handley ^135^ | Automated telephone self-management support, that is, interactive telephone technology to provide surveillance and patient education combined with nurse care management for patients with diabetes | Usual care | weekly, rotating automated (pre-recorded) telephone calls in their native language for 9 months (39 weeks). | 12 |
| Hanssen ^223^ | A structured, nurse-led intervention encompassing reactive and proactive telephone follow-up after discharge for patients with acute myocardial infarction | Usual care | 8 phone calls | 18 |
| Henderson ^165^ | Community based telehealth (Whole Systems Demonstrator telehealth questionnaire study) intervention for patients with long-term conditions | Usual care | Telehealth - no further support | 12 |
| Hermiz ^178^ | Home visits post discharge, involving detailed assessment plus verbal & written care plan, plus preventive GP care for patients | Usual care |  | 3 |
| Hernandez ^179^ | Specialist team discharge assessment, pharmacological therapy plus education, & home hospitalisation visits, including reinforcement of action plan by physician | Conventional inpatient/ discharge care, | 4.5 hours | 8 |
| Holland ^224^ | Drug review and symptom self-management and lifestyle advice intervention by community pharmacists for patients with heart failure, post discharge | Usual care | Two pharmacist home visits at 2 weeks after discharge and 6-8 weeks after discharge = 2 hours | 6 |
| Hurley ^151^ | Combined (group + individual) rehabilitation involving consisted of 12 supervised sessions (twice weekly for 6 weeks) by physiotherapist for patients with chronic knee pain | Usual care | 12 sessions twice weekly for 6 weeks= 12 hrs | 6 |
| Hurley ^151^ | Group rehabilitation involving consisted of 12 supervised sessions (twice weekly for 6 weeks) by physiotherapist | Usual care | 12 sessions twice weekly for 6 weeks= 12 hrs | 18.3 |
| Hurley ^151^ | Individual rehabilitation involving consisted of 12 supervised sessions (twice weekly for 6 weeks) by physiotherapist | Usual care | 12 sessions twice weekly for 6 weeks= 12 hrs | 18.3 |
| Jansa ^261^ | Trained in the management of a telecare system - the GlucoBeep system (device, patient software, unit & professional software) – in replacement of face-to-face outpatient appointments for patients with type 1 diabetes and poor metabolic control | Usual care | 1 teaching-training session in using the telecare system | 6.12 |
| Jayadevappa ^225^ | Transcendental meditation, a behavioural intervention for stress reduction, plus educational group-based sessions, for African Americans with congestive heart failure | Health education | 7 initial 1hr-30 ms sessions + 9 further meetings= 8 hours the least | 6 |
| Jerant ^287^ | Homing in on Health, a Chronic Disease Self-Management Program variant, peer-led, face-face | Usual care | home based one-to one 6 weekly sessions lasting approximately 2 hours each delivered by trained peers with chronic conditions= 6 sessions= 12 hrs | 12 |
| Jerant ^287^ | Telephone-based in on Health, a Chronic Disease Self-Management Program variant | Usual care | home based one-to one 6 weekly sessions lasting approximately 2 hours each delivered by trained peers with chronic conditions= 6 sessions= 12 hrs | 12 |
| Jessep ^159^ | Integrated rehabilitation programme (Enabling Self-Management and Coping with Arthritic Knee Pain though Exercise -knee pain) that combined exercise, patient education, self-management and coping strategies | Usual care | 10 1-h physiotherapist led sessions within 5 weeks + 1 review session at 4 months. | 4.12 |
| Johnson ^254^ | Group program led by physiotherapists involving exercise and education using a cognitive behavioural therapy approach for patients with persistent disabling low back pain | Usual care | eight 2-hour group sessions over a 6-week period | 3, 9, 15 |
| Jolly ^226^ | Programme to coordinate preventive care led by specialist liaison cardiac nurses which sought to improve communication between hospital and general practice and to encourage general practice nurses to provide structured follow up for patients with MI & angina | Usual care | At least 3 phone call specialist cardiac liai son nurses to practices | 12 |
| Jolly ^137^ | Post discharge, home-based, cardiac rehabilitation program (the Birmingham Rehabilitation Maximisation Study) including exercise, relaxation, education and lifestyle counselling, home visits & telephone contact | Centre-based rehabilitation | Visit at home | 3,6,12, 24 |
| Irvine ^134^ | University of East Anglia Impaired Fasting Glucose program, including both diet & group-based physiotherapist-led exercise components; peer support group & telephone support to prevent type 2 diabetes in patients with impaired fasting glucose | Usual care | 17.5 Hours to deliver training seminars; 21 Minutes of calls per participant (no other info) | 8 |
| Karjalainen ^255^ | Mini-intervention, based on features of a light mobilization program & graded activity program, with physiotherapist and physician support for patients with subacute low back pain | Usual care | 1,5 hrs consultation with physician and physiotherapist | 3,6,12,24 |
| Karjalainen ^255^ | Identical to mini-intervention group; Visit to patients worksite by a nurse, physiotherapist and physician, work supervisor to assess work conditions & provide support & feedback sent to GP | Usual care | 1,5 hrs consultation with physician and physiotherapist + worksite visit | 3,6,12,24 |
| Kasper ^227^ | Multidisciplinary outpatient management program consisting of phone calls, a therapeutic plan, and one nurse visit in patients with heart failure at high risk of hospital readmission | Usual care | 11 calls + 6 monthly visit | 6 |
| Katon ^273^ | Multifaceted, stepped collaborative care intervention, targeting the patient and the physician and the process of care using collaborative management by a psychiatrist and a primary care physician for persistently depressed primary care patients | Usual care | 2 sessions with psychiatrist (1st 50 ms and 2nd 25 mins)= 1 hr and 15mins. | 18 |
| Katon ^274^ | Multifaceted intervention targeting the patient and the physician and the process of care using collaborative management by a psychiatrist and a primary care physician for patients with panic disorder | Usual care | 2 sessions with psychiatrist (1st 1hr and 2nd 30 mins)+ at least 4 phone calls= 1 hr and 50 mins | 12 |
| Katon ^275^ | Provided access to a depression care manager supervised by a psychiatrist and primary care physician offered education support for antidepressant medication and problem solving therapy for late-life depression | Usual care | 1 initial session + 6 sessions for problem-sovling therapy+ 18 meetings/calls=5 hours? | 24 |
| Katon ^143^ | CBT and pharmacotherapy collaborative care intervention for panic disorder delivered in primary care by a mental health therapist. | Usual care | 6 sessions within 3 months, 6 telephone sessions between 3-12 months | 12 |
| Katon ^142^ | Medically supervised nurse, working with each patient’s primary care physician, provided guideline-based, collaborative care management of multiple diseases. | Advanced usual care | 18 sessions in primary care in twelve months | 12 |
| Kauppinen ^126^ | Intensive education programme, including use of inhaled drugs, PEF monitoring & including self-management plan for newly diagnosed patients with asthma | Conventional education | 150 mins | 36 |
| Kennedy ^164^ | Lay-led, generic, self-care support programme, the Expert Patients Programme was developed by researchers at Stanford University in the USA for patients with long-term conditions | Usual care | 6 weekly 2.5 hrs sessions with 8-10 participants | 6 |
| Khdour ^127^ | Hospital pharmacy-led, structured, disease medicine management program, including action plan & motivational interviewing (Cost-effectiveness) | Usual care | 1 hour, plus 40 mins of telephone calls, plus 30 mins outpatient visit = 2 hours, 10 mins | 12 |
| Ko ^180^ | Early outpatient pulmonary rehabilitation exercise programme after hospitalization for acute exacerbations | Usual care | thrice per week for 8 weeks and spent 2 h in each session | 3, 6, 9, 12 |
| Koff ^128^ | Proactive integrated care , multi component intervention for patients; 4 components 1) disease-specific education; 2) teaching of SM; 3) enhanced communication with coordinators; 4) remote home monitoring (‘Health Buddy’) | Usual care | 30 mins introductory session; 20 mins per day Health Buddy Sustem session; 9 hours daily monitoring of patients | 3 |
| Koehler ^228^ | Physician-led remote telemedical management that used portable devices for ECG, blood pressure, and body weight measurements connected to a personal digital assistant that sent automated encrypted transmission via cell phones to the telemedical care for patients with chronic heart failure | Usual care | 4 follow-up visits | 26 |
| Kroenke ^288^ | Combined pharmacological therapy and pain self-management program, consisting of a nurse care manager (depression care management team, developed for primary care patients with depression & musculoskeletal pain) | Usual care | optimized pharmacotherapy, 6 sessions of a pain self-management (PSM) program over 12 weeks and, a continuation phase of therapy for 6 months which included 2 phone calls= 6 sessions + 2 calls= 3hrs and 10 mins the minimum | 6, 12 |
| Kwok ^229^ | Community nurse-supported hospital discharge programme involving community nurse visits pre & post discharge for older patients with chronic heart failure | Usual care | 1 pre-discharge nurse meeting + 9 home visits= 4hrs and 30 mins the least | 6 |
| Lahdensuo ^181^ | Guided self-management group, including personal education, physiotherapeutic counselling, & diary recordings for patients with asthma | Traditional treatment | 150 mins + daily diary recordings | 12 |
| Lee ^182^ | Nursing home care protocol of individualised care following hospitalisation in older nursing home patients with chronic obstructive pulmonary disease | Usual care | 1 hour plus weekly CM nurse visits (30 mins) for first month (2 hours); CM nurse visits (30 mins) at monthly intervals (6 months = 3 hours) plus telephone support calls (15 mins) in between visits (6 months = 1 hour, 15 mins) Total 7 hours & 15 mins | 6 |
| Levitt ^276^ | Illness management and recovery group-based programme, including case management, psychiatric treatment and medication, for patients with serious mental illness who were receiving supportive housing services | Waiting list | 41 supporting sessions | 12 |
| Levy ^183^ | Structured education sessions by Emergency room based specialist nurses, using self-management plan, for emergency room attendance for asthma | Usual care | 2 hours | 6 |
| Lewin ^230^ | Home-based self-help rehabilitation programme (‘the heart manual’) for post infarct patients that included education, a home-based exercise programme, and a tape-based relaxation and stress management programme. | Usual care | 4 contacts (either phone or face to face) with the facilitator (physician) | 12 |
| Lewin ^138^ | Brief home-based cognitive behavioural rehabilitation programme for patients receiving an implantable cardioverter-defibrillator introduced before implantation, with brief telephone contacts with nurse | Usual care | 4 contacts (either phone or face to face) with the facilitator-nurse | 6 |
| Linton ^256^ | Primary care, group CBT intervention, focusing on preventing long-term disability by changing patients with spinal pain behaviours and beliefs so they can cope better with their problems. | Information-pamphlet | 6 2-hr group sessions over 6 weeks | 12, 60 |
| Linton ^256^ | A packet of information once a week for 6 weeks | Information enhanced | 6 2-hr group sessions over 6 weeks | 12, 60 |
| Lopez-Cabezas ^231^ | Multifactorial educational intervention carried out by a pharmacist involved receiving information about the disease, drug therapy, diet education, and active telephone follow-up in patients with heart failure | Usual care | Initial meeting with physician + 6 monthly phone calls and 3 calls once in two months= 9 contacts (per 10min)+1 (30 mins)= 2 hours | 12 |
| Man ^184^ | Outpatient pulmonary rehabilitation program, multidisciplinary team-led with exercise & educational components | Usual care | 2 hours per class = 32 hours | 3 |
| Mancuso ^185^ | Multicomponent, behavioural based, Emergency department education program (workbook, behavioural contract, telephone calls, physiologic feedback) for patients with asthma | Instruction/ PF training | 2 hours & 10 mins (15mins of calls x 8 weeks + 10 mins to make contract) | 12 |
| Markle-Reid ^232^ | Specialized, evidence-based, interprofessional team approach to community-based stroke rehabilitation | Usual care | Indvidualised plan with three initial appointments and home visits (unclear the intensity | 12 |
| McBeth ^257^ | Telephone-delivered CBT, involving patient-centred assessment, by developing a shared understanding and formulation of problem, and identified patient-defined goals for patients with chronic widespread pain | Usual care | 1 initial assessment (45-60 minutes), 7 weekly sessions (each 30-45 minutes long), and 1 session 3 months and 1 session 6 months after randomization= 5hrs 15 mins= 11sessions. | 6, 9 |
| McBeth ^257^ | A leisure-facility– and gym based exercise program consistent with American College of Sport Medicine guidelines for improving cardiorespiratory fitness | Usual care | Following 1 induction session, patients were offered 6 fitness instructor–led monthly appointments for program reassessment= 7 sessions=3,5 hrs | 6, 9 |
| McBeth ^257^ | The above two combined | Usual care | 18 sessions= 18hrs and 45 mins | 6, 9 |
| McDonald ^233^ | Multidisciplinary care involving inpatient and outpatient medical care, education and close telephone and clinic follow-up for patients with heart failure | Usual care | At least 3 inpatient education visits from specialist nurse, 12 weekly phone calls and 2 visits to HF. | 3 |
| McLean ^187^ | Enhanced pharmaceutical care, including teaching of asthma self-management, medication usage & provision of asthma action plan, delivered by local community, experienced pharmacists | Usual care | 7 1-hour appointments with a pharmacist | 7 |
| McGeoch ^186^ | Provision of written self-management plan (action plan) & patient initiated medication administered in primary care | Usual care | 1 hour | 12 |
| McGowan ^262^ | Community peer-led group based self-management program with a focus on action planning, follow-up and problem solving for patients with type 2 diabetes | Usual care | 2.5 hours x 6 weeks | 0 |
| McWilliam ^289^ | Health promotion education therapy, individualized, led by nurses post discharge, for chronically ill older patients | Usual care | 10 weekly home visits by nurse = 10 hrs (mean 10.55 hrs) | 5, 12 |
| CPMMPT ^210^ | 12-month intervention comprised an initial consultation with a community pharmacist to review appropriateness of therapy, compliance, lifestyle, social and support issues | Usual care | At least one pharmacist consultation and further consultations based on the need | 12 |
| Mejhert ^234^ | Nurse based outpatient management programme and pharmacotherapy intervention for elderly patients with heart failure | Usual care | Regular visits of patients to outpatient clinic | 18 |
| Meijer ^152^ | Return-to-work, outpatient multidisciplinary treatment programme with psychological and physical sessions for patients with upper extremity musculoskeletal disorders | Usual care | 13 full days (from 9.00 to 17.00 hours), 5 return-to-work sessions and 1 feedback session= 62 sessions, 82 hrs | 2,6,12 |
| Moffett ^258^ | Exercise classes led by a physiotherapist that included strengthening exercises for all main muscle groups, stretching exercises, relaxation session, and brief education on back care, utilising elements of CBT, for patients with lower back pain in primary care. | Usual care | 8 sessions over 4 weeks= 4 hrs | 6.12 |
| Monninkhof ^129^ | Educational self-management outpatient program including a fitness programme, guidelines for self-treatment of exacerbations, and a self-management education course | Usual care | 10 hours (education component), plus 1.5 hours (x104 physiotherapist sessions) = 156 hours | 12 |
| Morcillo ^235^ | Single home-based educational intervention nurse-led, after hospital discharge, which included education and self-management advice for heart failure patients | Usual care | 2 hours nurse visit at home | 6 |
| Moudgil ^188^ | Individually-based, asthma education & optimisation of drug therapy program | Usual care | 120 mins | 4 |
| Murphy ^236^ | Complex intervention involving tailored care plans for practices (practice based training in prescribing and behaviour change, administrative support, quarterly newsletter), and tailored care plans for patients (motivational interviewing, goal identification, and target setting for lifestyle change) with reviews every four months at the practices for secondary prevention of heart disease in primary care | Usual care | 1 initial meeting with GP, 1 phone call from GP, consulatations ever 4 months= 7 meeting + 1phone calls= 4 hours | 24 |
| Murray ^237^ | Pharmacist-led intervention on medication compliance, involving multidisciplinary team, for patients with heart failure, with low health literacy & limited resources. | Usual care |  | 12 |
| Naylor ^238^ | Transitional care intervention, involving discharge planning & home follow-up protocol, delivered by advanced practice nurses for older adults hospitalized with heart failure | Usual care | daily visits during hospitalization, 8 home visits | 3 |
| Niemstro ^160^ | Combined manipulative treatment, stabilizing exercises, and physician consultation for patients with chronic low back pain | Physician consultation | 4 1-hr sessions over four weeks= 4 hrs | 5, 12, 24 |
| Ninot ^189^ | Supervised hospital-based exercise program, plus self-management education sessions | Usual care | 16.5 hours (plus 45 mins = x3 telephone follow up 2x per week post intervention) | 12 |
| Nucifora ^239^ | Nurse-led education programme, included pre-discharge patient education, post-discharge facilitated telephone communication and follow-up outpatient visits with an internist for patients with heart failure | Usual care | 1 half-hour visit during hospital, 1 phone call after discharge, 3 doctor home visits= 2 hour and 15 mins | 6 |
| Nunez ^251^ | Therapeutic education and functional readaptation program for patients with musculoskeletal diseases involving the lower limbs, designed to improve pain and functional disability and to increase patient disease self-management (based on social learning theory) | Usual care | 2 individual visits lasting about 30 min at first week and at 3 months, and 2 group sessions of about 90 min in weeks 3 and 4, for a maximum of 10-12 patients= 4 sessions, 4 hrs | 3.9 |
| Ojeda ^240^ | Post discharge intervention programme for patients with heart failure involving patient education, consultation with the cardiologist and monitoring in the Heart Failure Unit | Usual care | 1 education session prior to discharge + 6 clinic visits= 7 sessions= 3,5 hours at least | 12 |
| Patel ^153^ | Arthritis SM programme plus education booklet in primary care patients with osteoarthritis of the hips or knees, or both, and pain, or disability | Education booklet | 6 weekly group sessions of 2 ½ hours each= 9 hrs | 12 |
| Penn ^277^ | Community-based, therapist-led, group CBT, including emotional support & counselling components for patients with schizophrenia auditory hallucinations severity | Enhanced supportive therapy | 12 weekly sessions=6 hours | 6 |
| Penn ^278^ | The Graduated Recovery Intervention Program for patients with first episode psychosis; involved four phases delivered by a therapist: (1) engagement and wellness management; (2) substance use; (3) persistent symptoms; and (4) functional recovery. | Treatment as usual | 12 sessions up to 36 | 12 |
| Peters ^259^ | Multidisciplinary inpatient pain management programme, CBT-based | Usual care | 4 days per week for 4 weeks | 9-12 |
| Peters ^259^ | Multidisciplinary outpatient pain management programme, education based | Usual care | 9, weekly, 2 h sessions at the hospital= 18 hrs | 9-12 |
| Pinnock ^191^ | Nurse-delivered, routine review by telephone of patients with asthma in primary care | Usual care | telephone call by nurse | 3 |
| Pilotto ^190^ | Nurse-run asthma clinics, including the provision of an action plan, in primary care | Usual care | 3 nurse follow-up visits to review the inhaler technique and encourage patients to develop action plans | 6, 9 |
| Price ^192^ | Use of personal action plans through implementation of adjustable dosing in asthma patients | Fixed dosing normal management |  | 3 |
| Pyne ^144^ | Rural-based, collaborative care depression intervention; Stepped-care model for treatment involving an off-site depression care team (nurse depression care manager, clinical pharmacist, psychiatrist) to make treatment recommendations via electronic medical record, & communication via telephone & computerized decision support software | Usual care |  | 12 |
| Ramachandran ^241^ | Telephone-based disease management programme involving interactive sessions with the patient with heart failure and spouse, and a telephonic helpline and regular telephone calls | Usual care | 2 initial face-to-face sessions (1 hour) and 25 phone calls (25x5=125mins)=3 hours and 5 mins | 6 |
| Rea ^193^ | Disease management program, including a care plan and coordination of care | Usual care | 12 visits to PN (6 hours) plus 4 visits to GP (2 hours) plus two home visits (1 hour) = 9 hours | 12 |
| Reynolds ^277^ | Transitional discharge model to support patients with mental health conditions discharged from admission wards to community living; two components included peer support, and overlap of inpatient and community staff relationship & coordination of care | Usual care | At least 4 home visits by inpatient nurses + peer support | 5 |
| Rich ^242^ | Nurse-directed, multidisciplinary intervention consisted of comprehensive education for the patient with congestive heart failure and family, a prescribed diet, social-service consultation and planning for an early discharge, a review of medications, and intensive follow-up | Usual care |  | 1 |
| Richardson ^290^ | A rehabilitation multi-component intervention for patients with chronic conditions was delivered by a physiotherapist and occupational therapist in primary care setting and included collaborative goal setting for rehabilitation needs, chronic disease self-management workshop, referral to community programs and a web-based education programme | Usual care | Collaborative goal setting for rehabilitation needs, individual treatment as needed, a six-week group SM workshop | 6, 9 |
| Riegel ^243^ | Nurse-led telephone case management, using a decision-support software program (‘At Home with Heart Failure’) for Hispanic patients with heart failure, post discharge | Usual care | 13.5 telephone contacts +8,6 family contacts + 4.6 nurse consultations with other professionals= 26.6 contacts= approximately 3 hours | 6 |
| Ries ^194^ | Telephone maintenance program following rehabilitation program in patients with chronic lung disease | Usual care | Weekly telephone calls (15 mins x52 weeks = 13 hours); Monthly reinforcement sessions = 1.5 hours supervised exercise, 1 hour topic review, 0.5 hours social time (3 hours) Total = 16 hours | 24 |
| Rivera ^280^ | Consumer-assisted providers of case management which involved provision of social support through matching peer staff with consumers with severe mental impairment | Usual care | standard care plus + peer support | 12 |
| Rivera ^280^ | Clinic-based case management which mainly included provision of support via professional | Usual care | standard care plus + peer support | 12 |
| Roberts ^291^ | Individualized 1-hr counselling meetings (1-10)conducted by nurses over a 6 month period for patients with chronic conditions | Usual care | 1-10 meetings lasting 1 hr | 6,12 |
| Roberts ^291^ | Individualized telephone counselling by nurses | Usual care | calls (5-10 mins) every two weeks for the first two months and then every month for four months= 80 mins the minimum | 6,12 |
| Roelfs ^161^ | Short intervention involving wearing a lumbar support for home care workers when/ anticipated to experience chronic back pain | Usual care | no session | 12 |
| Ryan ^195^ | Mobile phone supported self-monitoring, including transmission of symptoms, drug use, & PF with feedback according to a plan for patients with asthma | Usual care | Twice daily recordings per week | 6 |
| Schermer ^130^ | Guided, individual, SM from primary care physicians, including educational tools for patient & physician, & PF monitor in patients with asthma | Usual care |  | 24 |
| Schwarz ^244^ | Telemonitoring by an advanced practice nurse | Usual care | telemonitoring + advance nurse contacts | 6 |
| Seto ^245^ | Mobile phone-based telemonitoring system to record daily weight, blood pressure readings & assess symptoms, plus telephone technical support, for heart failure management | Usual care | 1 instruction sesssion | 6 |
| Sevick ^154^ | aerobic exercise training intervention consisted of a 3-month facility-based program and a 15-month home-based program | Health education | 3 60-min sessions per week for 3 months (n= 36 sessions)+ 4 home visits + 6 phone calls + 3 phone calls + 8 phone calls= 57 contacts = (36 hrs + 2 hrs +2,5 hrs)= 40 hrs | 3 |
| Sevick ^154^ | Resistance exercise training intervention consisted of a 3-month facility-based program and a 15-month home-based program | Health education | 3 60-min sessions per week for 3 months (n= 36 sessions)+ 4 home visits + 6 phone calls + 3 phone calls + 8 phone calls= 57 contacts = (36 hrs + 2 hrs +2,5 hrs)= 40 hrs | 3 |
| Seymour ^196^ | Outpatient, post exacerbation pulmonary rehabilitation program following hospitalisation | Usual care | 2 hours, twice-weekly, exercise & education sessions | 3 |
| Shelledy ^197^ | In-home asthma disease management program, respiratory therapist-led, involving asthma education for patient & family, educational tools and care plan | Usual care | 5 hours | 6 |
| Simon ^132^ | Diabetes glycaemic education and monitoring trial for patients with type 2 diabetes; Less intensive group = use of blood glucose meter + advice to contact GP for interpretation | Usual care | 15 mins (assessment visit) + 5 mins (record 3 values, 2 days per week) + 5 mins (diary entry) over 9 months; 6 days of nurse training x 5 weeks | 12 |
| Simon ^132^ | Diabetes glycaemic education and monitoring trial; More intensive group = use of blood glucose meter + training to interpret results | Usual care | 15 mins (assessment visit) + 5 mins (record 3 values, 2 days per week) + 5 mins (diary entry) over 9 months; 6 days of nurse training x 5 weeks | 12 |
| Simon ^147^ | Depression management programme which included patient education, antidepressant pharmacotherapy in primary care, telephone monitoring and psychiatric consultation if needed | Usual care | 8 primary physician visits= 4 hours + possible psychiatric consultations | 12 |
| Simon ^281^ | Depression relapse prevention program involving: systematic patient education, psychoeducational visits with a depression prevention specialist, shared decision-making regarding maintenance pharmacotherapy, and telephone and mail monitoring of medication adherence and depressive symptoms | Usual care | 2 visits with depression specialist+ 4 telephone monitoring contacts + 4 personalized emailings | 12 |
| Simon ^282^ | Nurse care manager provided 2-year systematic intervention program, including: structured group psychoeducational program, telephone monitoring of mood symptoms and medication adherence, feedback to treating mental health providers, facilitation of appropriate follow-up care, and as-needed outreach and crisis intervention | Usual care | 24 phone calls+ 48 weekly groups sessions= 72= 12 hrs +4=16 hrs | 24 |
| Simon ^145^ | Telephone care management intervention included outreach calls for monitoring and support, feedback to treating physicians, and care coordination for patients with depression | Usual care | up to 5 brief telephone calls | 12 |
| Simon ^145^ | The care management plus telephone psychotherapy intervention added an 8-session structured CBT program with up to 4 additional calls for reinforcement. | Usual care | 12 telephone calls +8 sessions= 5 hrs | 12 |
| Sinclair ^246^ | Home-based intervention for older cardiac patients consisted of home visits after hospital discharge by nurse who encouraged compliance with and knowledge of treatment regimen, offered support and guidance about resuming daily activities | Usual post-discharge care | 2 nurse home visits (no duration is reported)= 1 hour | 3 |
| Sisk ^247^ | Nurse-led intervention focused on specific self-management problems plus scheduled follow-up calls for minority communities with heart failure | Usual care | 1 appointment with nurse, additonal calls (no information on the number, co-ordination with patient's clinician | 12 |
| Soler ^198^ | Short educational program included visits to specialised nurse-led clinic & short educational program (but no SM plan) | Usual care | Monthly visits to clinic (1 hour) plus educational session (30 mins) total = 12 hours, 30 mins | 12 |
| Solomon ^252^ | Arthritis Self-Management Program course, incorporating educational materials such as SM plan, in primary care | Arthritis handbook only | 6 weekly sessions, each about 2 hours in duration, led by a trained facilitator= 12 hrs | 4 |
| Strong ^162^ | Lay-led, self-care, group-based intervention in reducing impairment and activity limitations in patients with moderate back pain in primary care | Usual care | 4 weekly group sessions | 3,6,12 |
| Strong ^162^ | Psychologist-led self-care interventions in reducing impairment and activity limitations in patients with moderate back pain | Usual care | 2 two-hr group sessions, 1 45-minindividual session, and a brief (3 min) follow-up phone call=4 sessions= 4hrs and 47 mins | 3,6,12 |
| Sundberg ^199^ | Computerised, educational, interactive program involving questions & graphics for young adults with asthma, followed by discussion with asthma nurse at outpatient clinic | Usual care | 1 hour | 12 |
| Swerissen ^292^ | Chronic disease management programme for patients with chronic illness from Vietnamese, Chinese, Italian & Greek backgrounds | Usual care | six weekly sessions of 2.5 h in duration using the Chronic Disease Self-Management Workshop—Leaders Manual | 6 |
| Taylor ^139^ | Home-based cardiac rehabilitation, nurse facilitated, self-help program (‘the Heart Manual’) | Hospital rehabilitation | 2 face to face sessions and 4 phone calls (5-10mins)= 100 mins | 9 |
| Thomas ^155^ | Home-based exercise program consisted of quadriceps strengthening plus telephone contact and aerobic exercise taught in a graded program for patients with knee pain | No intervention | 4 30 min visits during the initial 2 months and 1 visit every 6 months= 8 visits= 4hrs | 24 |
| Trento ^263^ | Physician-led lifestyle intervention by group care, including education sessions plus optional individual care for patients with type 2 diabetes | Usual care | 34 mins + 45 mins = 1 hour, 19 mins (plus 24 mins for elective individual visits) | 51 |
| Turkington ^283^ | Mental health nurse-led brief CBT designed to improve patients’ understanding, to develop their coping skills and help them to take more control over their schizophrenia | Usual care | 6 sessions within 2-3 months | 12 |
| van der Meer ^131^ | Internet-based self-management programme, including electronic personal action plan, group & on-line education for patients with asthma | Usual primary care, face-to-face |  | 12 |
| Varma ^248^ | Structured pharmaceutical education program on disease and its treatment and lifestyle changes. | Usual care | 1 education session | 12 |
| Wakabayashi ^200^ | Integrated care program including educational sessions & treatment & management plan, according to patient score on Lung Information Needs Questionnaire for older patients with COPD | Education based on LINQ | 3 hours | 12 |
| Wakefield ^249^ | Nurse-delivered telehealth-facilitated post-discharge support program with self-efficacy components, for patients with heart failure | Usual care | 14 phone calls= 60 mis | 3 |
| Wakefield ^249^ | Video-health-facilitated post-discharge support program | Usual care | 14 phone calls= 60 mis | 3 |
| Watson ^201^ | SM plan plus SM booklet | Usual care | 1 hour | 6 |
| Weinberger ^156^ | Interventions consisted of providing information & differed in mode of delivery: Telephone only group was phoned monthly and/or scheduled visits in Clinic | Clinic visits or no intervention | monthly phone calls + clinic visits for 1 year | 12 |
| Whitehurst ^157^ | Brief pain management program (BPM) physiotherapy-led targeting psychosocial risk factors for patients with low back pain in primary care | Physical therapy programme | 40-minute assessment/treatment session, plus up to 6 subsequent 20-minute treatment sessions= 7 sessions= 2hrs and 40 mins | 3, 12 |
| Whooley ^284^ | Case-finding for depression intervention; Primary care physicians notified of depression score (Geriatric Depression Scale) & offered psycho-educational sessions led by nurse | Usual care | 6 weekly sessions + 1 booster session= 3,5 hrs the lower | 24 |
| Willmott ^250^ | Intervention included expressive writing about patients’ thoughts and feelings in relation to having had an infarct | Attention control | only instructions were given during the nurse visit | 5 |
| Wolf ^264^ | Dietician-led lifestyle case management individual & group support sessions, for obese patients with type 2 diabetes in primary care | Usual care | 4 hours of group sessions + 6 hours of small group sessions + 15mins brief telephone calls | 12 |
| Wootton ^293^ | multidisciplinary intervention to improve the coordination of primary acute and residential care services | Usual care |  | 12 |
| Yilmaz ^202^ | Out-patient clinic, special education programme for patients with asthma | Usual care |  | 36 |
| Yoon ^203^ | Brief, group-based, single session, education program for adults with asthma, including inhaler use, adjust medication dosage using a treatment plan | Delayed intervention | 3 hours | 10 |
